# A Shared Role for RBF1 and dCAP-D3 in the Regulation of Transcription with Consequences for Innate Immunity

**DOI:** 10.1371/journal.pgen.1002618

**Published:** 2012-04-05

**Authors:** Michelle S. Longworth, James A. Walker, Endre Anderssen, Nam-Sung Moon, Andrew Gladden, Margarete M. S. Heck, Sridhar Ramaswamy, Nicholas J. Dyson

**Affiliations:** 1 Department of Molecular Genetics, The Lerner Research Institute, Cleveland Clinic, Cleveland, Ohio, United States of America; 2 Massachusetts General Hospital Cancer Center and Harvard Medical School, Charlestown, Massachusetts, United States of America; 3 Center for Human Genetic Research, Massachusetts General Hospital, Boston, Massachusetts, United States of America; 4 Department of Biology, Developmental Biology Research Initiative, McGill University, Montreal, Canada; 5 Department of Genetics, The University of Texas MD Anderson Cancer Center, Houston, Texas, United States of America; 6 Centre for Cardiovascular Science, Queen's Medical Research Institute, University of Edinburgh, Edinburgh, United Kingdom; Univeristy of Arizona, United States of America

## Abstract

Previously, we discovered a conserved interaction between RB proteins and the Condensin II protein CAP-D3 that is important for ensuring uniform chromatin condensation during mitotic prophase. The *Drosophila melanogaster* homologs RBF1 and dCAP-D3 co-localize on non-dividing polytene chromatin, suggesting the existence of a shared, non-mitotic role for these two proteins. Here, we show that the absence of RBF1 and dCAP-D3 alters the expression of many of the same genes in larvae and adult flies. Strikingly, most of the genes affected by the loss of RBF1 and dCAP-D3 are not classic cell cycle genes but are developmentally regulated genes with tissue-specific functions and these genes tend to be located in gene clusters. Our data reveal that RBF1 and dCAP-D3 are needed in fat body cells to activate transcription of clusters of antimicrobial peptide (AMP) genes. AMPs are important for innate immunity, and loss of either dCAP-D3 or RBF1 regulation results in a decrease in the ability to clear bacteria. Interestingly, in the adult fat body, RBF1 and dCAP-D3 bind to regions flanking an AMP gene cluster both prior to and following bacterial infection. These results describe a novel, non-mitotic role for the RBF1 and dCAP-D3 proteins in activation of the *Drosophila* immune system and suggest dCAP-D3 has an important role at specific subsets of RBF1-dependent genes.

## Author Summary

The retinoblastoma protein (pRB) is a tumor suppressor protein known for its ability to repress transcription of E2F-dependent genes and induce cell cycle arrest. We have previously shown that RB proteins in *Drosophila* and human cells interact with the Condensin II subunit, CAP-D3, in an E2F-independent manner. Condensins promote condensation of chomosomes in mitosis. Our previous studies suggested that the *Drosophila* pRB and CAP-D3 homologs, RBF1 and dCAP-D3, co-localize on DNA and may share a function in cells that never undergo mitosis. In this study, we show that one non-mitotic function shared between RBF1 and dCAP-D3 is the regulation of many non-cell-cycle-related, clustered, and cell-type-specific transcripts including a conserved family of genes that are important for the immune response in the fly. In fact, results show that normal levels of dCAP-D3 and RBF1 expression are necessary for the ability of the fly to clear infection with human bacterial pathogens. This work demonstrates that dCAP-D3 proteins can regulate a unique subset of RBF1-dependent transcripts *in vivo* and identifies a novel role for both RBF1 and dCAP-D3 protein in activation of innate immune genes, which may be conserved in human cells.

## Introduction

The RB family proteins (pRB, p130 and p107 in humans; RBF1 and RBF2 in *Drosophila*) co-ordinate changes in gene expression. Understanding the types of programs that these proteins regulate is important because of the unequivocal link between the inactivation of RB proteins and human cancer. Mutation of the retinoblastoma tumor susceptibility gene (*RB1*) is the rate-limiting step in the genesis of retinoblastoma and over 90% of human tumors exhibit reduced pRB function [1], [2].

RB family members are best-known for their roles in the regulation of E2F-dependent transcription. E2F-controlled genes are needed for cell proliferation and RB proteins suppress the expression of these targets during G0 and G1 of the cell cycle [3]. In addition, RB proteins are also important for the regulation of genes that are not involved in cell cycle progression. For example, osteoblast differentiation is modulated by pRB through its interaction with Runx2 [4]; in muscle cells, pRB promotes the expression of muscle-specific differentiation markers, enabling these cells to irreversibly exit the cell cycle [5]–[7]; in *Drosophila*, RBF1 cooperates with the Hippo pathway to maintain photoreceptor differentiation, independent of dE2F1 activity [8]. Such E2F-independent functions may help to explain why the inactivation of RB proteins can have very different consequences in different cellular contexts. However, many of the E2F-independent activities of RB proteins are not well-understood. At present, it is unclear if pRB has different activities in different cell types, or whether there is a yet-to-be discovered, general process that allows RB proteins to activate or repress the expression of variable sets of genes in different cell types.

Recent studies have suggested that pRB family members may impact the organization of higher-order chromatin structures, in addition to their local effects on the promoters of individual genes [9]. Mutation of pRB causes defects in pericentric heterochromatin [10] and RBF1 is necessary for uniform chromatin condensation in proliferating tissues of *Drosophila* larvae [11]. Part of the explanation for these defects is that RBF1 and pRB promote the localization of the Condensin II complex protein, CAP-D3 to DNA both in *Drosophila* and human cells [11]. Depletion of pRB from human cells strongly reduces the level of CAP-D3 associated with centromeres during mitosis and causes centromere dysfunction [12].

Condensin complexes are necessary for the stable and uniform condensation of chromatin in early mitosis [13]–[16]. They are conserved from bacteria to humans with at least two types of Condensin complexes (Condensin I and II) present in higher eukaryotes. Both Condensin I and II complexes contain heterodimers of SMC4 and SMC2 proteins that form an ATPase which acts to constrain positive supercoils [17], [18]. Each type of Condensin also contains three specific non-SMC proteins that, upon phosphorylation, stabilize the complex and promote ATPase activity [14], [19], [20]. The kleisin CAPH and two HEAT repeat containing subunits, CAP-G and CAP-D2 are components of Condensin I, while the kleisin CAP-H2 and two HEAT repeat containing subunits, CAP-G2 and CAP-D3, are constituents of Condensin II.

Given the well-established functions of Condensins during mitosis, and of RBF1 in G1 regulation, the convergence of these two proteins was unexpected. Nevertheless, mutant alleles in the non-SMC components of Condensin II suppress RBF1-induced phenotypes, and immunostaining experiments revealed that RBF1 displays an extensive co-localization with dCAP-D3 (but not with dCAP-D2) on the polytene chromatin of *Drosophila* salivary glands [11]. This co-localization occurs in cells that will never divide, suggesting that Condensin II subunits and RBF1 co-operate in an unidentified process in non-mitotic cells. In various model organisms, the mutation of non-SMC Condensin subunits has been associated with changes in gene expression [21]–[24] raising the possibility that dCAP-D3 may affect some aspect of transcriptional regulation by RBF1. However, the types of RBF1-regulated genes that might be affected by dCAP-D3, the contexts in which this regulation becomes important, and the consequences of losing this regulation are all unknown.

Here we identify sets of genes that are dependent on both *rbf1* and *dCap-D3*. The majority of genes that show altered expression in both *rbf1* and *dCap-D3* mutants (larvae or adults) are not genes involved in the cell cycle, DNA repair, proliferation, but are genes with cell type-specific functions and many are spaced within 10 kb of one another in “gene clusters”. To better understand this mode of regulation we have investigated the effects of RBF1 and dCAP-D3 on one of the most highly misregulated clusters which includes genes coding for antimicrobial peptides (AMPs). AMPs are produced in many organs, and one of the major sites of production is in the fat body. Following production in the fat body, AMPs are subsequently dumped into the hemolymph where they act to destroy pathogens [25]. RBF1 and dCAP-D3 are required for the transcriptional activation of many AMPs in the adult fly. Analysis of one such gene cluster shows that RBF1 and dCAP-D3 bind directly to this region and that they bind, in the fat body, to sites flanking the locus. RBF1 and dCAP-D3 are both necessary in the fat body for maximal and sustained induction of AMPs following bacterial infection, and RBF1 and dCAP-D3 deficient flies have an impaired ability to respond efficiently to bacterial infection. These results identify dCAP-D3 as an important transcriptional regulator in the fly. Together, the findings suggest that RBF1 and dCAP-D3 regulate the expression of clusters of genes in post-mitotic cells, and this regulation has important consequences for the health of the organism.

## Results

### RBF1 and dCAP-D3 regulate many of the same genes during the later stages of the *D. melanogaster* life cycle

**Figure 1:**
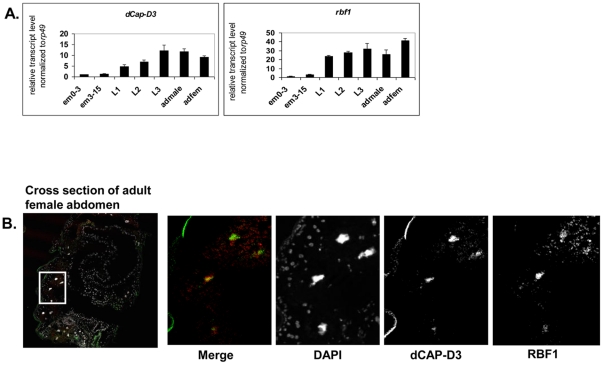
RBF1 and dCAP-D3 are highly expressed at later stages of development and co-localize in adult tissues. A) qRT-PCR for *rbf1* transcript levels and *dCap-D3* transcript levels in wild type *Drosophila* embryos aged for 0–3 hours (em0–3), embryos aged for 3–15 hours (em3–15), first instar larvae (L1), second instar larvae (L2), third instar larvae (L3), adult males (admale) or adult females (adfem) demonstrate high expression levels in the later life cycle stages. B) Immunostaining for RBF1 and dCAP-D3 in cryosections of adult female flies indicates co-localization in large nuclei of cells present underneath the cuticle. Images presented are a magnification of the area highlighted by the white box in the first image.

Our previous data demonstrated that RBF1 co-localizes extensively with dCAP-D3 on polytene chromatin of non-dividing cells, leading us to hypothesize that the two proteins may co-operate to regulate transcription. To begin to test this idea, we first identified the stages of fly development where RBF1 and dCAP-D3 were most highly expressed. qRT-PCR using primers for *dCap-D3* and *rbf1* was performed on cDNA generated from various stages of the *Drosophila* life cycle (Figure 1A). The results demonstrate that both genes are transcribed at the highest levels in late third instar larval and adult stages. Concordantly, immunostaining for dCAP-D3 and RBF1 in cryosections of the abdomens of wild type flies confirmed that both proteins are highly expressed in the adult and that they are both present in the nuclei of many cells in normal adult tissues (Figure 1B).

**Figure 2:**
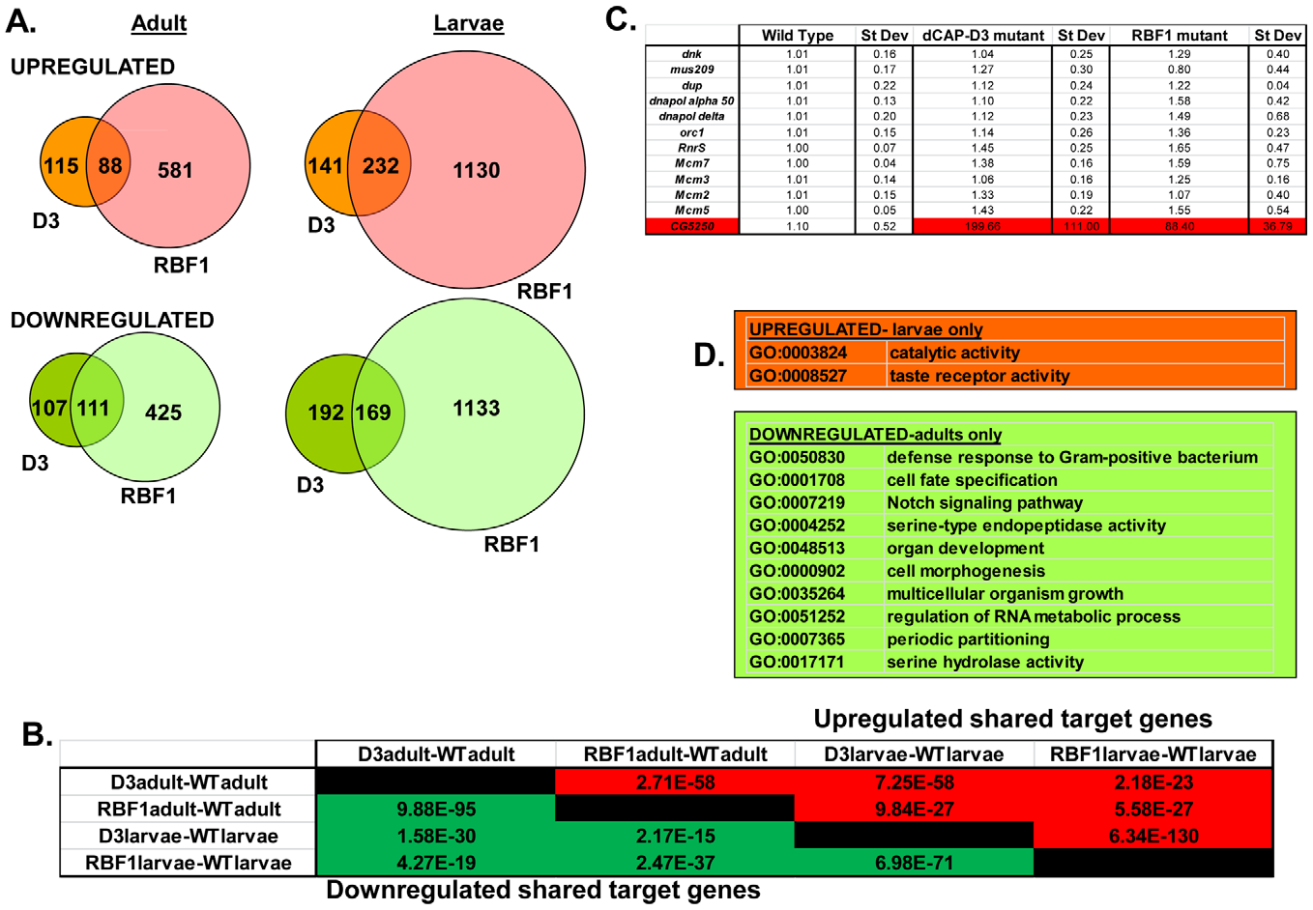
RBF1 and dCAP-D3 regulate many of the same transcripts in the fly. RNA was isolated from *rbf1* mutant and *dCap-D3* mutant female third instar larvae and adult flies. cDNA was hybridized to Nimblegen 385 k whole genome arrays. A) Venn diagrams show the numbers of RBF1, dCAP-D3 or RBF1/dCAP-D3 shared target genes which exhibited at least a 2 fold log change in expression with a p value of ≤0.15. Genes significantly upregulated in the mutant flies are shown in red while genes significantly downregulated are shown in green. B) P values for shared RBF1 and dCAP-D3 target genes indicate that RBF1 and dCAP-D3 regulate a significant number of the same genes in both adults and larvae. The numbers above the diagonal represent p-values for upregulated shared subsets and are colored red while the numbers below the diagonal represent p-values for downregulated shared genes and are colored green. C) qRT-PCR analyses of 12 E2F targets shows that the majority of RBF1/dCAP-D3 shared targets are not E2F targets. The one target that was significantly upregulated in dCAP-D3 and RBF1 mutant flies, *CG5250*, is highlighted in red. Results are the average of three independent experiments involving 10 female flies per genotype. D) Significant (p≤0.05) Gene Ontology (GO) groupings for shared target genes include defense response genes in the adult fly. The top box lists GO categories for upregulated shared genes in mutant larvae only, and the bottom box lists selected GO categories for downregulated shared genes in adults only. There were no significant GO groupings for upregulated shared target genes in adults or for downregulated shared target genes in the larvae.

Preliminary experiments showed that dCAP-D3 levels could influence the expression of very few of the previously identified RBF1-dependent transcripts. To gain a more complete understanding of the abundance and characteristics of RBF1/dCAP-D3 shared transcriptional targets, we carried out a microarray analysis of the entire *Drosophila melanogaster* genome and compared gene expression profiles of wild type, *dCap-D3* and *rbf1* mutant flies, at both the third instar larval and adult stages (Table S1). Since the null mutants are lethal, females expressing a transheterozygous combination of null and hypomorphic alleles were used for these experiments. The mutant flies used for microarray analysis expressed about 15% of wild type levels of each gene as judged by qRT-PCR and western blot (Figure S1). The microarray results revealed an extensive and highly significant overlap between RBF1 and dCAP-D3 regulated gene sets in both adults and larvae (Figure 2A and 2B). Shared target genes were evident in both upregulated and downregulated gene sets. Although some genes were mis-expressed in both larvae and adults, the majority of transcriptional changes were stage specific. The most highly significant p values for shared target gene sets were seen in upregulated larval genes (genes repressed by RBF1 and dCAP-D3 in the larvae, p≤6.34E-130) and downregulated adult genes (genes activated by RBF1 and dCAP-D3 in the adult, p≤9.88E-95) (Figure 2B). This suggests that RBF1 and dCAP-D3 may cooperate to repress specific programs during one stage of development and activate other programs in a later, more differentiated stage. Interestingly, at both stages, the genes dependent on both RBF1 and dCAP-D3 represented 15–17% of the total number of genes dependent on RBF1 in a given developmental stage and 47–55% of the total number of genes dependent on dCAP-D3 in a given developmental stage. Thus RBF1 appears to be important at close to half of the transcriptional targets of dCAP-D3.

### Characteristics of RBF1/dCAP-D3 shared target genes

We noticed that the lists of RBF1/dCAP-D3 shared target genes had two general properties. First, these genes are almost completely different from the lists of E2F-regulated genes that have been reported previously [26]. As expected, many of the targets that were upregulated in *rbf1* mutant larvae could be categorized as E2F target genes involved in DNA repair, DNA replication and continuation of the cell cycle (comparison to microarray data from [26] and GO analyses of rbf1 mutant larvae- data not shown). However, few if any, of these cell cycle/proliferation related genes were altered in the *dCap-D3* mutant flies (Figure 2C) suggesting that *dCap-D3* regulates a different subset of RBF1 dependent targets. In fact, less than 6% of dCAP-D3/RBF1 shared target genes in larvae were found to be bound by dE2F1 in dE2F1 ChIP-chip experiments (Korenjak et. al., unpublished data). Unexpectedly, many of the known E2F target genes did not show a significant increase in expression in *rbf1* mutant adults (Figure 2C). This may reflect cell-type specific differences in the requirement for RBF1. In support of this idea, qRT-PCR analysis of dissected tissues showed that few E2F-regulated genes were upregulated in ovaries of *rbf1* mutants, but many did show a significant increase in the rest of the carcass (Figure S2). However, even in the tissues where these E2F-regulated proliferation genes did increase in expression levels in *rbf1* mutant adults, these transcripts were not upregulated in tissues from *dCap-D3* mutant flies (Figure S2). We infer that dCAP-D3 is not a key factor at most of the well-characterized E2F regulated genes in either larvae or adults. While unlikely, it is a formal possibility that the remaining amounts of dCAP-D3 protein present in the hypomorphic mutant flies might be sufficient for the regulation of E2F targets, but not for other target genes.

Second, we noted that genes that are similarly dependent on RBF1 and dCAP-D3 tend to be clustered on the genome and are often positioned within 10 kb of one another (Table 1). To determine whether this was an unusual feature, we compared the frequency of RBF1/dCAP-D3shared target genes positioned within 10 kb of one another to hundreds of simulations of randomly chosen *Drosophila* genes (Table 1). The results showed that genes exhibiting increased expression in *rbf1* and *dCap-D3* mutant adults (ie. genes that are apparently repressed both by RBF1 and dCAP-D3 are 25 times more likely to be clustered. Genes that were downregulated in *rbf1* and *dCap-D3* mutant adults (i.e. genes apparently activated by both RBF1 and dCAP-D3) are 15 times more likely to be clustered. Clustering of shared target genes was also seen in the larvae, although the fold difference was greatly diminished (5 fold) for the activated genes. Overall, the clustering effect was 3–7 fold more prevalent in dCAP-D3 regulated genes than in RBF-regulated genes. By way of comparison, RBF1/dCAP-D3 shared target genes in the larvae exhibited a much greater degree of clustering than the larval genes regulated by Hop or Nurf301, two other well-known chromatin remodeling proteins shown to regulate clusters of genes [27]. A list of the actual groupings of clustered genes is presented in Table S2.

**Table 1:** RBF1 and dCAP-D3 tend to regulate clusters of genes.

*Gene Set*	*Ratio for upregulated* * ∧	*Ratio for downregulated* * ∧
Whole adult dCAP-D3 mut	12.42	11.68
Whole adult RBF1 mut	3.93	4.41
Whole adult shared targets	25.00	15.87
Whole larvae dCAP-D3 mut	14.2	11.59
Whole larvae RBF1 mut	2.60	2.03
Whole larvae shared targets	22.58	3.68
Whole larvae Hop mut**	1.91	1.30
Whole larvae Nurf301 mut**	1.55	1.30

*: The ratio of observed clustering to expected clustering for the lists of differentially expressed genes between mutant and wild -type organisms (fdr<0.15, log2 fc>0.1) shows that RBF1 and dCAP-D3 shared target genes are 6–10 fold more likely to be present in clusters. Chromosomal clustering is calculated as the number of pairs of genes within 10,000 bp among the differentially expressed genes. The expected number is the average clustering of 500 random gene lists of the same length as the corresponding list of differentially expressed genes.

**: Raw data for Hop and Nurf301 mutant larvae was obtained from supplemental data files found in [27].

**∧:** False Discovery Rates for all ratios presented were <0.05.

Although proliferation-related genes were missing, gene ontology (GO) classification of the RBF1/dCAP-D3 shared target genes revealed many significant categories in the lists of up- and downregulated genes in the adult, and for shared, repressed target genes in the larvae (Figure 2D, complete list for downregulated adult genes in Table S3). One of the most interesting GO categories represented in the adults were the defense response genes (GO:0050830). The fly relies on an innate immune system to defend against invading pathogens. This immune system is comprised of three major mechanisms: 1) phagocytosis, 2) induction of coagulation and melanization, and 3) production of Antimicrobial Peptides or AMPs.

Phagocytosis is a conserved mechanism that is often the primary cellular defense used by many organisms to engulf and destroy pathogens. In *Drosophila*, circulating blood cells called hemocytes phagocytose bacteria, fungi, and parasitic wasp eggs [28]. RBF1 and dCAP-D3 mutant adult microarray data was analyzed for changes in levels of 19 different genes reported to be involved in phagocytosis in *Drosophila* (Table S4). Of these 19 genes, 2 genes demonstrated significant changes in transcript levels in adults. *NimC1*, a gene expressed in plasmatocytes which make up 95% of *Drosophila* hemocytes, has been shown to be necessary for phagocytosis of bacteria [29], and was significantly upregulated in RBF1 and dCAP-D3 mutant adults. Embryonic and larval hematopoiesis depends on a number of transcription factors including Gcm [30], [31]. *Gcm* transcripts were demonstrated to be downregulated in both RBF1 and dCAP-D3 adults (Table S4). While adult hemocytes do display phagocytic properties, they do not differentiate into specialized cells upon immune challenge [32], [33], and it is therefore unlikely that misregulation of *gcm* in adults would affect phagocyte numbers.

In response to septic injury, proteolytic cascades are triggered which lead to coagulation and melanization. Reactive oxygen species formed during these processes, as well as the actual deposition of melanin, are thought to be toxic to microorganisms [34]. After scanning the literature for genes involved in coagulation and melanization, and then analyzing RBF1 and dCAP-D3 mutant adult microarray data for changes in transcript levels of these genes, it was determined that only one of the reported genes, *CG8193* was significantly increased in both RBF1 and dCAP-D3 mutant adults (Table S5). CG8193/PPO2 is thought to encode a phenol-oxidase constitutively expressed in crystal cells, a type of hemocyte cell involved in melanization [35]. However, overexpression of CG8193/PPO2 in cell lines or in flies does not induce constituitive melanization [36], nor did we see any evidence of melanotic lesions in RBF1 or dCAP-D3 mutant adults.

**Figure 3:**
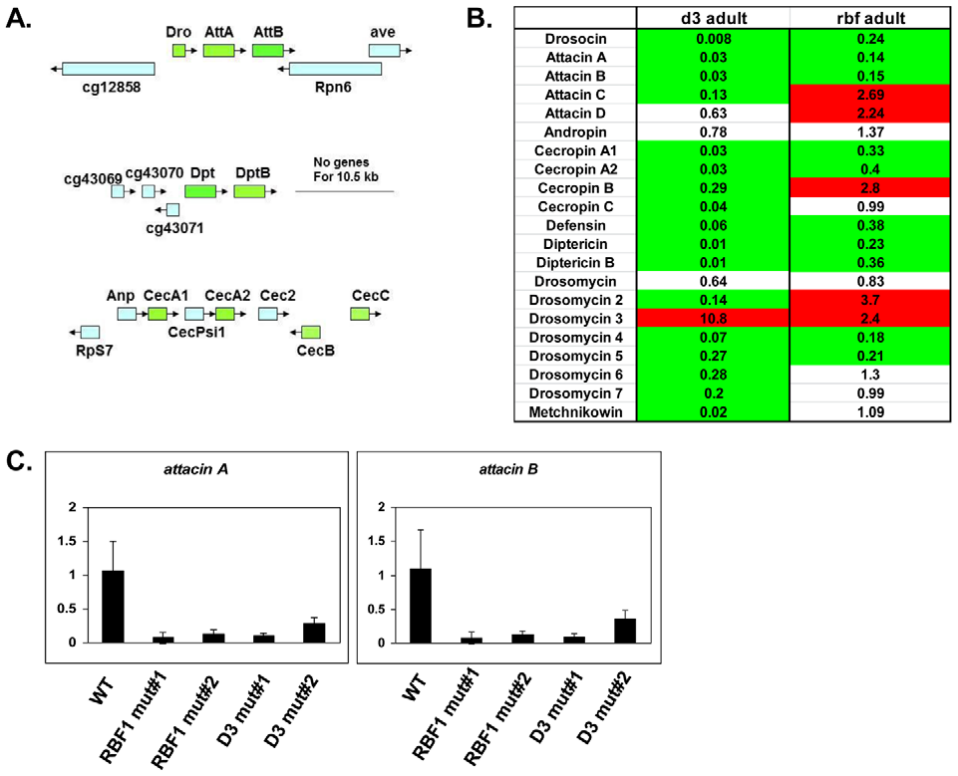
RBF1 and dCAP-D3 activate basal transcript levels of genes coding for Antimicrobial Peptides (AMPs). A) Graphic depictions of three separate AMP loci. The *Attacin* and *Diptericin* loci are located on Chromosome 2R at 51C1 and 55F8, respectively. The *Cecropin* locus is located on Chromosome 3R at 99E2. Genes within each locus are drawn in correct orientation to one another but are not drawn to scale. Genes colored in green are downregulated in dCAP-D3 mutant adult carcasses dissected of ovaries and most are also downregulated RBF1 mutants. Genes colored in blue remain unchanged in dCAP-D3 and RBF1 mutants. B) qRT-PCR analyses of transcript levels for 21 AMPs in female adult bodies (N = 10) with ovaries dissected shows that dCAP-D3 and RBF1 each regulate a much larger number of AMPs than originally indicated by the microarray results. Genes significantly upregulated in the mutants are highlighted in red and genes significantly downregulated in the mutants are highlighted in green. Transcript levels were normalized to tubulin 84B. All results had false discovery rates ≤0.05. C) qRT-PCR analysis of cDNA from wild type (WT/*w^1118^*), *rbf1* mutant #1 (*rbf1^120a^/rbf1^Δ14^*), *rbf1* mutant #2 (*rbf1^120a^/rbf1^120a^*), *dCap-D3* mutant #1 (*dCap-D3^c07081^/dCap-D3^Δ25^*) and *dCap-D3* mutant #2 (*dCap-D3^c07081^/dCap-D3^c07081^*) female adult whole flies confirms that two AMP target genes are regulated by both RBF1 and dCAP-D3, regardless of mutant genotype. Transcript levels were normalized to *tubulin 84B* mRNA levels.

Several of the genes in the adult, downregulated GO category of “defense response to Gram positive bacteria” (Figure 2D and Table S3) fall into a family of proteins known as Antimicrobial Peptides or AMPs. In fact, two of these genes, *AttA* and *AttB*, represented some of the most highly deregulated targets in the mutant adults. Upon closer inspection of the microarray data, it was revealed that many other AMP genes were also deregulated in dCAP-D3 and/or RBF1 mutant adults, however their p-values were just below the confidence level. In addition, many of the AMP genes are present in clusters and located immediately next to one another in the genome (Figure 3A), making them an enticing group of genes for further study.

### AMPs are shared targets of RBF1 and dCAP-D3 in the adult fat body

To confirm that the transcription of AMPs was indeed dependent on both RBF1 and dCAP-D3, qRT-PCR analysis was performed using cDNA generated from *dCap-D3* or *rbf1* transheterozygotes (using whole female mutant flies whose ovaries had been dissected) (Figure 3B). Results showed that 17 of the 21 AMPs tested were downregulated in the *Cap-D3* mutants and 10 of those genes were similarly dependent on RBF1. qRT-PCR for AMPs performed on different allelic combinations of *rbf1* and *Cap-D3* mutants gave similar results (Figure 3C).

AMPs constitute one of the major defense mechanisms against bacterial and/or fungal infection in the fly [25], [37], [38]. They are produced in various adult tissues but one of the main organs responsible for their production is the fat body. Once produced in the fat body, AMPs are secreted into the hemolymph where they destroy or inhibit growth of pathogens [39].

**Figure 4:**
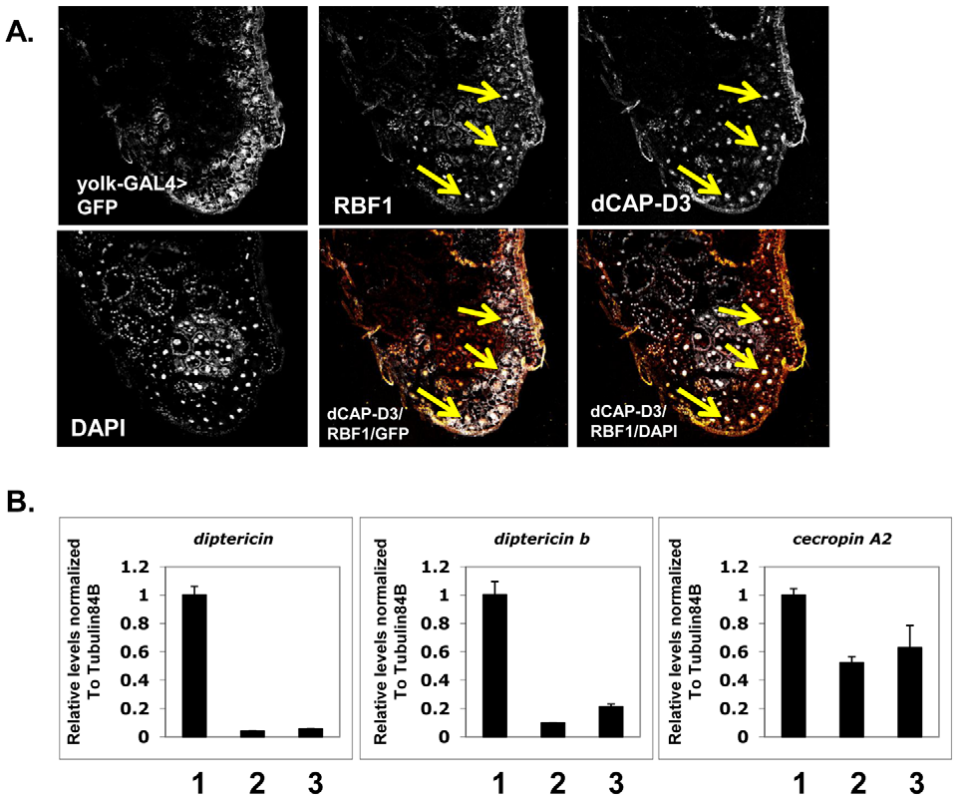
Basal AMP transcript levels are activated by RBF1 and dCAP-D3 specifically in the fat body. A) Immunofluorescence analysis of RBF1 and dCAP-D3 performed on cryosections of adult female flies expressing GFP under the control of the fat body specific *yolk-GAL4* driver indicates that RBF1 and dCAP-D3 co-localize in the nuclei of fat body cells. Yellow arrows highlight fat body cells. B) qRT-PCR analysis of cDNA from 1) flies expressing driver alone (*yolk-GAL4/+;+;+*), 2) flies expressing *rbf1 dsRNA (yolk-GAL4;+;UAS-rbf1 dsRNA)* in the fat body cells and 3) flies expressing *dCAP-D3 dsRNA (yolk-GAL4;+;UAS-dCAP-D3 dsRNA)* in the fat body cells shows significant decreases in AMP levels. For each genotype, N = 10.

We set out to test the hypothesis that RBF1 and dCAP-D3 regulate AMP genes in the adult fat body. First, we examined whether RBF1 and dCAP-D3 are expressed in this cell type. The *yolk-GAL4* driver was used to express GFP in adult fat body cells, effectively marking this cell type in green. Combined immunostaining for RBF1 and dCAP-D3 localization in cryosections of adult wild type abdomens revealed a strong staining for both RBF1 and dCAP-D3 in the nuclei of adult fat body cells (Figure 4A, yellow arrows). *yolk-GAL4* has been characterized to drive expression in *Drosophila* at approximately 2–5 days post eclosure [40], making it possible to drive expression of transgenes after the majority of fly development has occurred. The staining for RBF1 and dCAP-D3 in the adult abdomens was specific, as *yolk-GAL4* driven expression of dsRNAs directed against RBF1 and dCAP-D3 specifically abrogated staining of their respective targets in fat body cells, without dramatically altering gross tissue morphology (Figure S3).

Next, we measured the changes in expression of AMPs in animals where *yolk-GAL4* driven expression of dsRNAs had reduced the expression of either RBF1 or dCAP-D3 in the fat body. qRT-PCR of cDNA from whole adult females showed a significant decrease in the expression of multiple AMP genes including *diptericin*, *diptericin B* and *Cecropin A2* (Figure 4B) in the knockdown flies. Interestingly, the fold change in transcript levels for *diptericin* was comparable to the changes seen in *dCap-D3* and *rbf1* mutant animals. These results suggest that the yolk-GAL4-expressing cells are a primary site of constitutive *diptericin* expression in adult flies and that in these cells, RBF1 and dCAP-D3 are both needed to drive the basal expression levels of specific AMPs.

### Regulation of an AMP cluster by RBF1 and dCAP-D3 is direct and dynamically changes over the course of bacterial infection

**Figure 5:**
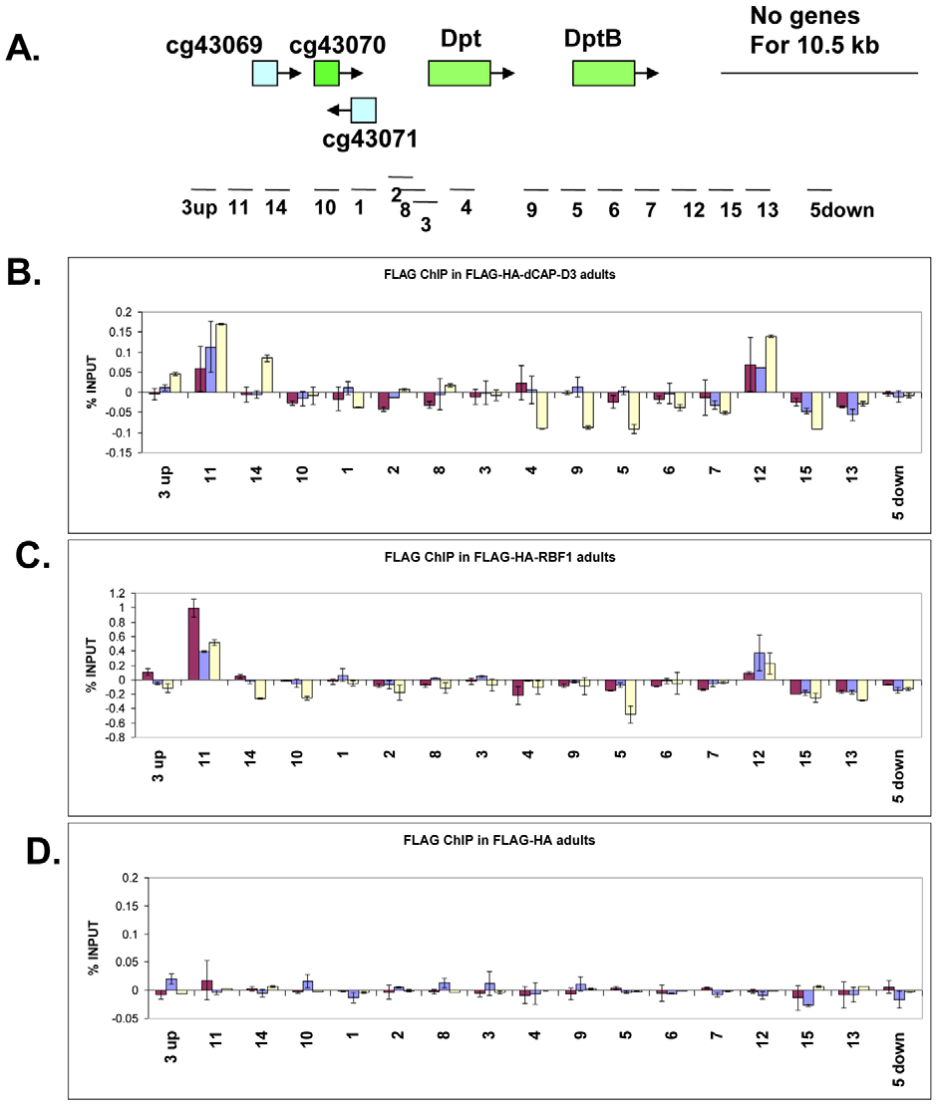
RBF1 and dCAP-D3 bind to an AMP locus *in vivo*. A) Graphic representation of the locus on which Chromatin Immunoprecipitation (ChIP) for RBF1 and dCAP-D3 in the adult fat body was performed. Genes highlighted in green are activated by both RBF1 and dCAP-D3 in the fat body. Positions of primer sets used are listed under the diagram of the locus. B) Chromatin immunoprecipitation for FLAG protein in female adult flies expressing FLAG-HA-dCAP-D3 in the fat body (*yolk-GAL4/+; +; UAS-FLAG-HA-dCap-D3/+*) demonstrates that the *diptericin* locus is a direct target of dCAP-D3. ChIP signal corresponding to FLAG-HA-dCAP-D3 binding in the absence of *Staphylococcus aureus* infection is colored in burgundy. ChIP signal corresponding to FLAG-HA-dCAP-D3 binding two and four hours after *S. aureus* infection is colored in blue and yellow, respectively. C) Chromatin immunoprecipitation for FLAG protein in female adult flies expressing FLAG-HA-RBF1 in the fat body (*yolk-GAL4/+; +; UAS-FLAG-HA-rbf1/+*) demonstrates that the locus is also a direct target of RBF1. Depiction of signal is as described in B. D) Chromatin immunoprecipitation for FLAG protein in female adult flies expressing FLAG-HA in the fat body (*yolk-GAL4/+; +; UAS-FLAG-HA/+*) demonstrates minimal non-specific binding of tag alone at the locus. Depiction of signal is as described in B.

AMP genes can be regulated by multiple transcription factors [41]–[44]. We sought to determine, therefore, whether transcriptional regulation of these genes by RBF1 and dCAP-D3 was direct. For our ChIP analysis we focused on *diptericin* and *diptericin B*; two AMP genes that are situated within 1200 bp of one another (Figure 5B), that have well characterized promoters [45]–[47], and whose basal expression was dependent on both RBF1 and dCAP-D3 in the fat body (Figure 3B and Figure 4B). In addition, the basal transcript levels of at least one other gene in the region, *CG43070*, was found to be significantly activated by both RBF1 and dCAP-D3 (Figure S4).

To study the binding of RBF1 and dCAP-D3 at the *diptericin* locus *in vivo*, transgenic fly lines were created which expressed N-terminally FLAG-HA tagged dCAP-D3 or N-terminally FLAG-HA tagged RBF1 under the control of the UAS promoter. These lines were then crossed to *yolk-GAL4/FM7* lines to create progeny in which the tagged protein was specifically expressed in the adult fat body. ChIP using FLAG antibody in FLAG-HA-dCAP-D3 expressing flies demonstrated that dCAP-D3 binds to two separate regions located approximately 3 kb upstream and 900 bp downstream of the *diptericin* locus (Figure 5A and red bars in Figure 5B). Since *diptericin* is strongly induced in response to bacterial infection, we examined the effect of infection with *S. aureus* on the binding of dCAP-D3 to the *diptericin* locus. Strikingly, dCAP-D3 binding to the upstream site significantly increased after *S. aureus* infection (compare red bars to yellow bars, Figure 5B).

ChIP for FLAG in FLAG-HA-RBF1 expressing flies indicated that RBF1 binds to the identical upstream and downstream regions of the *diptericin* locus as dCAP-D3 (red bars in Figure 5C). This binding was detected both before and after infection with *S. aureus* (blue and yellow bars in Figure 5C), but unlike the results for dCAP-D3 binding, RBF1 binding was most significant prior to infection. ChIP for FLAG protein in flies expressing the FLAG-HA construct alone showed almost no signal at any of the primer sets used in these experiments (Figure 5D). Taken together, ChIP results show that 1) RBF1 and dCAP-D3 can bind directly to an AMP gene cluster at identical binding sites, 2) that the binding sites flank the *diptericin* and *diptericin B* genes, and 3) dCAP-D3 binding increases when gene expression is induced in response to bacterial infection.

For comparison, we also performed ChIP for dCAP-D3 on the *CG5250* locus. *CG5250* was the one previously identified direct target of RBF1 [26] that we found to be repressed by RBF1 and dCAP-D3 and to be consistently upregulated in all tissues of *rbf1* and *dCap-D3* mutant animals (Figures S2 and S5A). ChIP using FLAG antibody in FLAG-HA-dCAP-D3 expressing flies demonstrated a small amount of binding in the open reading frame of *CG5250* (Figure S5B and S5C). This binding pattern obtained with the FLAG antibody closely resembled the ChIP signal found when a dCAP-D3 antibody was used to immunoprecipiate the endogenous dCAP-D3 protein expressed everywhere in the adult fly (Figure S5D).

**Figure 6:**
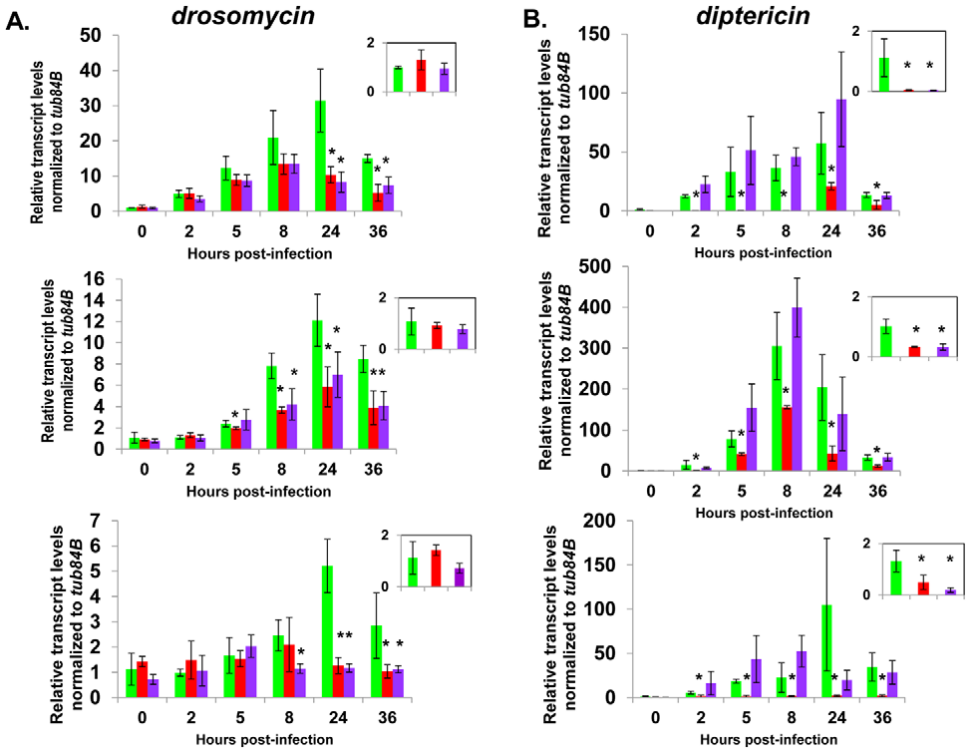
Complete AMP induction following bacterial infection depends on dCAP-D3 and RBF1. Adult female flies expressing RBF1 (purple) or dCAP-D3 (red) dsRNAs under the control of *yolk-GAL4* were infected with the Gram positive bacteria, *Staphylococcus aureus*. A) qRT-PCR analyses for transcript levels of the *Drosomycin* AMP gene in these flies show that while flies expressing GFP dsRNAs under the control of *yolk-GAL4* (green) undergo a large induction of AMPs at 8–24 hours post-infection, flies expressing dCAP-D3 or RBF1 dsRNA in the fat body fail to exhibit maximal, sustained induction. B) qRT-PCR analyses for transcript levels of the *Diptericin* AMP gene in these flies show that while flies expressing GFP or RBF1 dsRNAs under the control of *yolk-GAL4* (green) undergo a large induction of AMPs at 8–24 hours post-infection, flies expressing dCAP-D3 dsRNA in the fat body fail to exhibit maximal, sustained induction. The inset boxes in the upper right corner of each graph are a larger representation of the 0 hour timepoint and depict basal transcription levels. Asterisks emphasize statistical significance (p≤0.05) as determined by a students paired t-test. Three independent experiments are shown and results for each experiment are the average of three sets of five infected adults per genotype, per timepoint.

The ability of RBF1 and dCAP-D3 to regulate basal levels of AMP transcription prompted the question of whether these proteins were also necessary for the regulation of AMP transcription in response to bacterial infection. cDNA was generated from female adult flies expressing dCAP-D3 or RBF1 dsRNAs specifically in the fat body, at various time-points post-infection with *Staphylococcus aureus* (Figure 6). dsRNAs have been used successfully in the past to decrease *in vivo* expression levels of proteins involved in innate immunity and to study their effects on responses to bacterial infection [48].

qRT-PCR for AMPs indicated two types of transcriptional defects in the RBF1 and dCAP-D3 deficient flies. In agreement with our earlier results, basal transcript levels of *diptericin* were reduced as a result of deficiency for either protein (Figure 6B, inset boxes). Following infection, *diptericin* transcripts remained very low in the dCAP-D3 deficient tissue and induction was minimal and severely delayed in comparison to GFP dsRNA expressing “wild type” control flies. RBF1 deficiency, however, allowed normal induction of *diptericin* transcripts. *Drosomycin* is an AMP gene downstream of the Toll pathway, and it is strongly induced following infection with Gram positive bacteria or fungi [49]. qRT-PCR for levels of *Drosomycin* revealed a much different defect in expression. Neither dCAP-D3 nor RBF1 deficiency in the fat body had any effect on basal levels of *Drosomycin*, a result consistent with our microarray data from whole flies. However, both dCAP-D3 and RBF1 deficiency caused significant decreases in the maximal expression levels of *drosomycin* at 24 hours post-infection (Figure 6A).

### The biological response to bacterial infection in the fly requires dCAP-D3 and RBF1

**Figure 7:**
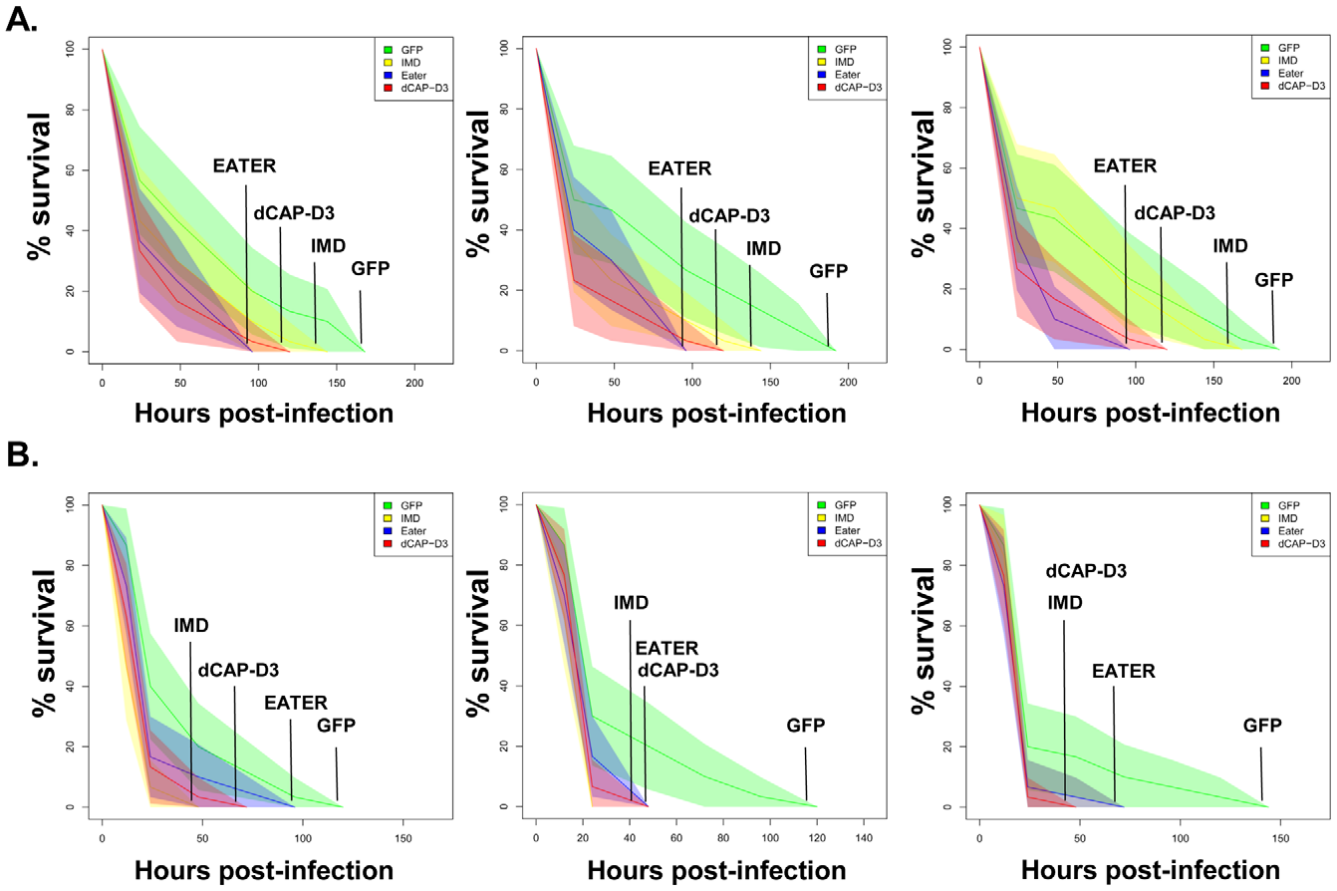
dCAP-D3 is necessary for a proper immune response to bacterial infection. Adult female flies expressing dCAP-D3 (red) dsRNAs under the control of *yolk-GAL4* were infected with the Gram positive bacterium, *Staphylococcus aureus* (A) or the Gram negative bacterium, *Pseudomonas aeruginosa* (B). Flies expressing GFP dsRNAs under the control of *yolk-GAL4* (green) were used as “wild-type” controls. Eater mutants which are defective in phagocytosis (blue) or flies expressing IMD dsRNAs which are compromised in a major innate immune signaling pathway (yellow) were used as positive controls. Results demonstrate that flies expressing reduced levels of dCAP-D3 in the fat body cells are more susceptible to either type of infection than wild type controls. Three independent experiments are depicted with results of each experiment shown as the average of three sets of 10 infected adults per genotype. These experiments were also performed using a sterile needle dipped in PBS to rule out death as a result of wounding and survival curves matched those of yolk-GAL4 expressing flies (data not shown). Results are presented as cox regression models with statistical significance (p≤0.05) represented as shaded areas above and below the curves.

**Figure 8:**
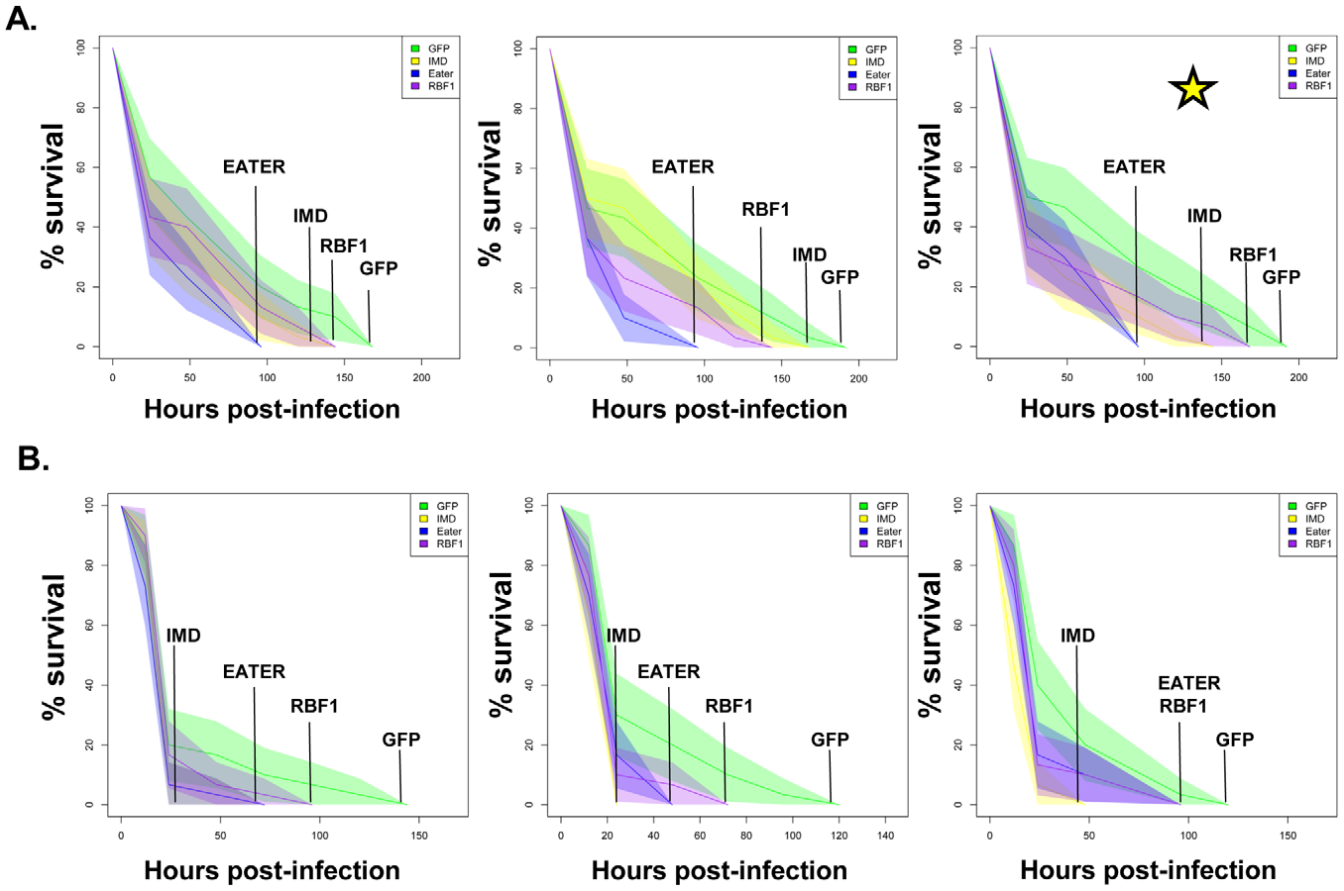
RBF1 is necessary for a proper immune response to Gram positive bacterial infection. Adult female flies expressing RBF1 (purple) dsRNAs under the control of *yolk-GAL4* were infected with the Gram positive bacterium, *Staphylococcus aureus* (A) or the Gram negative bacterium, *Pseudomonas aeruginosa* (B). Flies expressing GFP dsRNAs under the control of *yolk-GAL4* (green) were used as “wild-type” controls. Eater mutants which are defective in phagocytosis (blue) or flies expressing IMD dsRNAs which are compromised in a major innate immune signaling pathway (yellow) were used as positive controls. Results demonstrate that flies expressing reduced levels of RBF1 in the fat body cells are more susceptible to infection with Gram positive bacteria (A) than wild type controls. Three independent experiments are depicted with results of each experiment shown as the average of three sets of 10 infected adults per genotype. Results are presented as cox regression models with statistical significance (p≤0.05) represented as shaded areas above and below the curves. In the third experiment in (A), which is highlighted by a star, the survival endpoint becomes significant when the confidence level is changed to 90% (p≤0.10) instead of 95% (p≤0.05). These experiments were also performed using a sterile needle dipped in PBS to rule out death as a result of wounding and survival curves matched those of yolk-GAL4 expressing flies (data not shown).

Next, we tested whether the inefficient transcription of AMPs that results from decreased expression of RBF1 or dCAP-D3 has a significant effect on the ability of the fly to recover from exposure to pathogenic bacteria. Survival rates after infection with the Gram positive bacterium, *Staphylococcus aureus* (Figure 7A, Figure 8A) or with the Gram negative bacterium, *Pseudomonas aeruginosa* (Figure 7B, Figure 8B) were measured in five different genotypes: females expressing GFP dsRNAs under the control of the yolk-GAL4 driver (“wild-type controls”, *yolk-GAL4* driving expression of dCAP-D3 dsRNA in the fat body, *yolk-GAL4* driving expression of RBF1 dsRNA in the fat body, and positive control females which were either mutant for the Eater protein or expressing dsRNAs against the IMD protein. IMD is a major mediator of innate immune signaling in *Drosophila* [50]. Eater is a known phagocyctic receptor necessary for the response to infection with Gram positive bacteria [51]. We did not include data on flies expressing Eater dsRNAs under the control of yolk-GAL4, since Kocks et al [51] reported that Eater is not expressed in the fat body. In agreement with this, expression of Eater dsRNA in the fat body exhibited no changes in the ability of the fly to clear bacteria, while the Eater mutants described above showed a striking inability to phagocytize bacteria 5 hours following infection (data not shown and Figure 7). Following infection with *S. aureus*, both dCAP-D3 and RBF1 deficient flies were more susceptible to infection in comparison to flies expressing GFP dsRNAs (Figure 7A and Figure 8A). dCAP-D3 deficient flies were also more susceptible to infection with Gram negative bacteria, but this was not the case for RBF1 deficient flies, as their survival rates were not significantly decreased (Figure 7B and Figure 8B). These data demonstrate that acute knockdown of dCAP-D3 or RBF1 in the fat body of adult flies renders them more susceptible to bacterial infection, most likely due to inefficient transcription of AMP genes.

**Figure 9:**
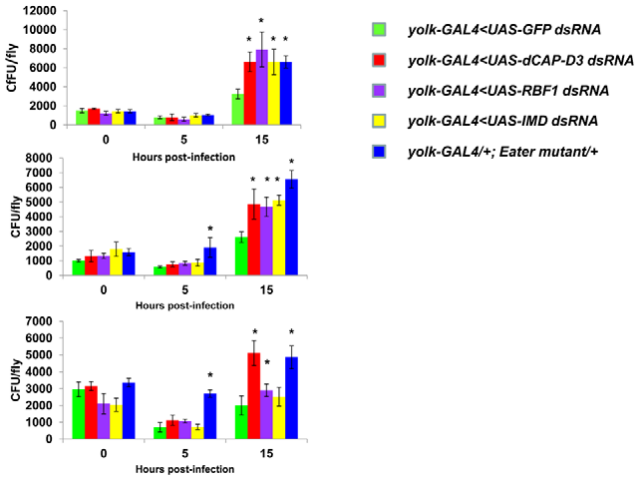
RBF1 and dCAP-D3 are necessary for the ability to clear bacteria *in vivo*. Adult female flies expressing RBF1 (purple) or dCAP-D3 (red) dsRNAs under the control of *yolk-GAL4* were infected with the Gram positive bacterium, *Staphylococcus aureus*. Flies expressing GFP dsRNAs under the control of *yolk-GAL4* (green) were used as “wild-type” controls. Eater mutants which are defective in phagocytosis (blue) or flies expressing IMD dsRNAs which are compromised in a major innate immune signaling pathway (yellow) were used as positive controls. Results demonstrate that at 15 hours following infection, flies expressing reduced levels of dCAP-D3 or RBF1 in the fat body cells exhibit increased numbers of bacteria in comparison to wild type controls. Three independent experiments are shown and results for each experiment are the average of three sets of three infected adults, per genotype, per timepoint. Asterisks emphasize statistical significance (p≤0.05) as determined by a students paired t-test.

Recently, a number of reports have identified genes whose mutation can reduce the ability of the fly to survive bacterial infection, without influencing the ability of the fly to clear bacteria [52]–[54]. These genes have been described as having affects not on the resistance mechanisms which exist in the fly, but on the tolerance mechanisms of the fly. Tolerance mechanisms limit the damage caused to the host by the infection, but do not actually limit the pathogen burden [55]. To determine whether loss of dCAP-D3 and/or RBF1 expression in the fat body did indeed result in diminished capacity of the fly to clear bacteria, we performed bacterial clearance assays and measured the number of bacteria present in the fly from 0–20 hours post-infection (Figure 9). Results showed that flies deficient for RBF1 or dCAP-D3 behave more like positive control flies deficient for IMD or Eater proteins, and exhibit significant increases in bacterial numbers at 15 hours post-infection with *S. aureus*. This suggests that RBF1 and dCAP-D3 most likely affect the resistance mechanisms (i.e. AMP transcription), and not the tolerance mechanisms of the fly.

### A second RBF family member, RBF2, regulates basal AMP transcription levels, but not induced levels, following infection

Since the observed defects in survival rates and AMP induction were not as severe for RBF1 deficient flies in comparison to dCAP-D3 deficient flies, we wondered whether the other *Drosophila* RBF member, RBF2, might compensate for loss of RBF1 activity. RBF2 has been shown to be upregulated upon depletion of RBF1, and co-regulates many genes with RBF1 as a part of the dREAM complex [26], [56], [57]. To address this question, we tested survival times and AMP induction in flies deficient for RBF2 in the fat body (yolk-GAL4<UAS-RBF2-dsRNA) or a combination of both RBF1 and RBF2 in the fat body (yolk-GAL4<UAS-RBF1 dsRNA, UAS-RBF2 dsRNA). The specific deficiencies in these flies were confirmed by qRT-PCR (Figure S6). qRT-PCR revealed that similar to the loss of RBF1 or dCAP-D3, loss of RBF2 or RBF1/RBF2 resulted in decreased basal transcript levels of *diptericin* but not *drosomycin* (Figure S7A and S7B, inset boxes). However, following infection with *S. aureus*, loss of RBF2 or RBF1/RBF2 did not cause decreased induction of either AMP transcript. In some cases, loss of both RBF1 and RBF2 actually resulted in an increase in *diptericin* transcription levels at 8 hours post infection. In response to infection with Gram positive bacteria (Figure S8A) or Gram negative bacteria (Figure S8B), RBF2 deficient or RBF1/RBF2 deficient flies did not exhibit any changes in survival rates that were significantly different from wild type control flies. These results demonstrate that RBF2 does regulate basal AMP transcript levels, but does not compensate for RBF1 in induction of AMP transcription in *Drosophila* following infection.

### The shared regulation of innate immune gene clusters by RBF1 and dCAP-D3 may be conserved in human cells

AMPs are conserved in many metazoans and play a very important role in fighting pathogens in barrier epithelial cells at mucosal surfaces [58]. pRB and CAP-D3 have been previously shown to interact physically and functionally in human cells [11]. Remarkably, and perhaps unexpectedly, the regulation of AMP genes by RB and CAP-D3 proteins may also be conserved in human cells. To determine whether pRB and CAP-D3 could regulate genes in human cells and, more specifically, whether the co-regulation of AMPs was conserved, siRNAs were used to decrease pRB and CAP-D3 expression in human Retinal Pigment Epithelial (RPE-1) cells and in premonocytic U937 cells. (Figure S9A and data not shown). qRT-PCR analyses of the levels of five different AMPs revealed that two AMPs (DEFB-3 and DEFA-1) were expressed in RPE-1 cells and both genes were significantly downregulated following the depletion of either pRB or CAP-D3 (Figure S9B). Interestingly, these genes are also located in a very large gene cluster, the *Defensin* locus, encompassing over 20 different AMPs. These data raise the possibility that the regulation of AMPs by CAP-D3 and pRB, and the ability of these proteins to regulate gene clusters, are properties that may be conserved in human cells.

## Discussion

In *Drosophila*, RB-family proteins are best known as transcriptional repressors of cell cycle and proliferation genes. Here we describe a different aspect of RB function and show that, together with the Condensin II protein dCAP-D3, RBF1 functions to regulate the expression of a large number of genes during *Drosophila* development. A surprising characteristic of RBF1/dCAP-D3 regulated genes is that they do not seem to be the classically repressed genes with functions in cell cycle progression, DNA damage and DNA replication. Instead, many RBF1/dCAP-D3-dependent genes are classified as being involved in cell-type specific functions and include genes that are involved in enzymatic cascades, organ development and cell fate commitment.

The idea that dCAP-D3 and RBF1 could cooperate to promote tissue development and differentiation is supported by the fact that both proteins are most highly expressed in the late stages of the fly life cycle, and accumulate at high levels in the nuclei of specific cell types in adult tissues. As an illustration of the cell-type specific nature of RBF1/dCAP-D3-regulation we show that dCAP-D3 and RBF1 are both required for the constituitive expression of a large set of AMP genes in fat body cells. The loss of this regulation compromises pathogen-induction of gene expression and has functional consequences for innate immunity. Interestingly, different sets of RBF1/dCAP-D3-dependent genes were evident in the gene expression profiles of mutant larvae and adults. Given this, and the fact that the gene ontology classification revealed multiple groups of genes, we suggest that the targets of RBF1/dCAP-D3-regulation do not represent a single transcriptional program, but diverse sets of cell-type specific programs that need to be activated (or repressed) in specific developmental contexts.

The changes in gene expression seen in the mutant flies suggest that RBF1 has a significant impact on the expression of nearly half of the dCAP-D3-dependent genes. This fraction is consistent with our previous data showing partial overlap between RBF1 and dCAP-D3 banding patterns on polytene chromatin, and the finding that chromatin-association by dCAP-D3 is reduced, but not eliminated, in *rbf1* mutant animals and RBF1-depeleted cells. Although we have previously shown that RBF1 and dCAP-D3 physically associate with one another [11], and our current studies illustrate the fact that they each bind to similar sites at a direct target, the molecular events that mediate the co-operation between RBF1 and dCAP-D3 remain unknown.

These results represent the first published ChIP data for the CAP-D3 protein in any organism. Although we have only examined a small number of targets it is interesting to note that the dCAP-D3 binding patterns are different for activated and repressed genes (compare Figure 5 and Figure S5). More specifically, dCAP-D3 binds to an area within the open reading frame of a gene which it represses (Figure S5C and S5D). However, dCAP-D3 binds to regions which flank a cluster of genes that it activates (Figure 5). Whether or not this difference in binding is true for all dCAP-D3 regulated genes will require a more global analysis.

Human Condensin non-SMC subunits are capable of forming subcomplexes *in vitro* that are separate from the SMC protein- containing holocomplex [59], but currently, the extent to which dCAP-D3 relies on the other members of the Condensin II complex remains unclear. We note that fat body cells contain polytene chromatin. Condensin II subunits have been shown to play a role in the organization of polytene chromatin in *Drosophila* nurse cells [60]. Given that RB proteins physically interact with other members of the Condensin II complex [11], it is possible that RBF1 and the entire Condensin II complex, including dCAP-D3, may be especially important for the regulation of transcription on this type of chromatin template.

A potentially significant insight is that the genes that are deregulated in both *rbf1* and *dCap-D3* mutants tend to be present in clusters located within 10 kb of one another. This clustering effect seems to be a more general feature of regulation by dCAP-D3, which is enhanced by RBF1, since clustering was far more prevalent in the list of dCAP-D3 target genes than in the list of RBF1 target genes.

We chose to focus our studies on one of the most functionally related families of clustered target genes that were co-dependent on RBF1/dCAP-D3 for activation in the adult fly: the AMP family of genes. AMP loci represent 20% of the gene clusters regulated by RBF1 and dCAP-D3 in adults. ChIP analysis of one such region, a cluster of AMP genes at the *diptericin* locus, showed this locus to be directly regulated by RBF1 and dCAP-D3 in the fat body and revealed a pattern of RBF1 and dCAP-D3-binding that was very different from the binding sites typically mapped at E2F targets. Unlike the promoter-proximal binding sites typically mapped at E2F-regulated promoters, RBF1 and dCAP-D3 bound to two distant regions, one upstream of the promoter and one downstream of the *diptericin B* translation termination codon, a pattern that is suggestive of an insulator function. We hypothesize that RBF1 and dCAP-D3 act to keep the region surrounding AMP loci insulated from chromatin modifiers and accessible to transcription factors needed for basal levels of transcription. The modEncode database shows binding sites for multiple insulator proteins, as well as GATA factor binding sites, at these regions. GATA has been previously implicated in transcriptional regulation of AMPs in the fly [61], and future studies of dCAP-D3 binding partners in *Drosophila* fat body tissue may uncover other essential activators. Additionally, the chromatin regulating complex, Cohesin, which exhibits an almost identical structure to Condensin [62]–[64], has been shown to promote looping of chromatin and to bind proteins with insulator functions [65], [66]. Therefore, it remains a possibility that Condensin II, dCAP-D3 may actually possess insulator function, itself. We would like to propose that dCAP-D3 may be functioning as an insulator protein, both insulating regions of DNA containing clusters of genes from the spread of histone marks and possibly looping these regions away from the rest of the body of chromatin. This would serve to keep the region in a “poised state” available for transcription factor binding following exposure to stimuli that would induce activation. In the case of AMP genes, which are made constituitively in specific organs at low levels [37], [67], [68], dCAP-D3 would bind to regions flanking a cluster, and loop the cluster away from the body of chromatin. Upon systemic infection, these clusters would be more easily accessible to transcription factors like NF-κB. If dCAP-D3 is involved in looping of AMP clusters, then it may also regulate interchromosomal looping which could bring AMP clusters on different chromosomes closer together in 3D space, allowing for a faster and more coordinated activation of all AMPs.

AMP expression is essential for the ability of the fly to recover from bacterial infection. Experiments with bacterial pathogens show that RBF1 and dCAP-D3 are both necessary for induction and maintenance of the AMP gene, *drosomycin* following infection, but only dCAP-D3 is necessary for the induction of the *diptericin* AMP gene. Similarly, survival curves indicate, that while dCAP-D3 deficient flies die more quickly in response to both Gram positive and Gram negative bacterial infection, RBF1 deficient flies only die faster in response to Gram positive bacterial infection. The differences seen between RBF1 and dCAP-D3 deficient flies in diptericin induction cannot be attributed to functional compensation by the other *Drosophila* RB protein family member, RBF2, since results show that loss of RBF2 or both RBF2 and RBF1 do not decrease AMP levels following infection. Since results demonstrate that RBF1 binds most strongly to an AMP cluster prior to infection and regulates basal levels of almost all AMPs tested, we hypothesize that RBF1 (and possibly RBF2) may be more important for cooperating with dCAP-D3 to regulate basal levels of AMPs. Reports have shown that basal expression levels of various AMPs are regulated in a gene-, sex-, and tissue-specific manner, and it is thought that constituitive AMP expression may help to maintain a proper balance of microbial flora and/or help to prevent the onset of infections [37], [68], [69]. In support of this idea, one study in *Drosophila* which characterized loss of function mutants for a gene called *caspar*, showed that *caspar* mutants increased constituitive transcript levels of diptericin but not transcript levels following infection. This correlated with increased resistance to septic infection with Gram negative bacteria [70], proving that changes in basal levels of AMPs do have significant effects on the survival of infected flies. Additionally, disruption of Caudal expression, a protein which suppresses NF-κB mediated AMP expression following exposure to commensal bacteria, causes severe defects in the mutualistic interaction between gut and commensal bacteria [71]. It is therefore possible that RBF1 and dCAP-D3 may help to maintain the balance of microbial flora in specific organs of the adult fly and/or be involved in a surveillance-type mechanism to prevent the start of infection. RBF1 deficient flies also exhibit defects in *Drosomycin* induction following Gram positive bacterial infection. Mutation to Drosophila GNBP-1, an immune recognition protein required to activate the Toll pathway in response to infection with Gram positive bacteria has been show to result in decreased *Drosomycin* induction and decreased survival rates, without affecting expression of *Diptericin* [72], [73]. Therefore, it is possible that inefficient levels of *Drosomycin*, a major downstream effector of the Toll receptor pathway, combined with decreased basal transcription levels of a majority of the other AMPs, would cause RBF1 deficient flies to die faster following infection with Gram positive *S. aureus* but not Gram negative *P. aeruginosa*.

Some dCAP-D3 remains localized to DNA in RBF1 deficient flies [11] and it is also possible that other proteins may help to promote the localization of dCAP-D3 to AMP gene clusters following infection. Given that dCAP-D3 regulates many AMPs including some that do not also depend on RBF1 for activation, and given that dCAP-D3 binding to an AMP locus increases with time after infection whereas RBF1 binding is at its highest levels at the start of infection, it may not be too surprising that dCAP-D3 showed a more pronounced biological role in pathogen assays involving two different species of bacteria.

Remarkably, and perhaps unexpectedly, the levels of both RBF1 and dCAP-D3 impact the basal levels of human AMP transcripts, as well. This indicates that the mechanism of RBF1/dCAP-D3 regulation may not be unique to *Drosophila*. It is striking that many of the human AMP genes (namely, the defensins) are clustered together in a region that spans approximately 1 Mb of DNA. It seems telling that both the clustering of these genes, and a dependence on pRB and CAP-D3, is apparently conserved from flies to humans. The fact that dCAP-D3 and RBF1 dependent activation of *Drosomycin* was necessary for resistance to Gram positive bacterial infection in flies suggests the same could also be true for the human orthologs in human cells. Human AMPs expressed by epithelial cells, phagocytes and neutrophils are an important component of the human innate immune system. Human AMPs are often downregulated by various microbial pathogenicity mechanisms upon infection [58], [74]–[76]. They have also been reported to play roles in the suppression of various diseases and maladies including cancer and Inflammatory Bowel Disease [77]. We note that the chronic or acute loss of Rb expression from MEFs resulted in an unexplained decrease in the expression of a large number of genes that are involved in the innate immune system [78]. In humans, the bacterium, *Shigella flexneri* was recently shown to down regulate the host innate immune response by specifically binding to the LXCXE cleft of pRB, the same site that we had previously shown to be necessary for CAP-D3 binding [11], [79]. An improved understanding of how RB and CAP-D3 regulate AMPs in human cells may provide insight into how these proteins are able to regulate clusters of genes, and may also open up new avenues for therapeutic targeting of infection and disease. Further studies of in differentiated human cells may identify additional sets of genes that are regulated by pRB and CAP-D3.

## Materials and Methods

### Fly strains

*W^1118^* flies were used as “wild type” controls for microarray experiments. Unless otherwise noted, the genotype of RBF mutants was a transheterozygous combination of *rbf1^Δ14^/rbf1^120a^* which was obtained by mating *rbf1^Δ14^/FM7*, *GFP* virgins to *rbf1^120a^/FM7*, *GFP* males at 18°C. Similarly, the genotype of CAP-D3 mutants was a transheterozygous combination of *dCAP-D3^Δ25^/dCAP-D3^c07081^* which was obtained by mating *dCAP-D3^Δ25^/CyO*, *GFP* virgins to *dCAP-D3^c07081^/CyO*, *GFP* males at 23°C. *yolk-GAL4/FM7c* flies were a kind gift of M. Birnbaum and the timing of expression driven by *yolk-GAL4* has been previously characterized in [40]. The RBF1, dCAP-D3, RBF2, and IMD dsRNA expressing strains were obtained from the VDRC and their transformant IDs were 10696, 29657, 100635, and 101834 respectively. UASt-FLAG-HA tagged strains were created by first amplifying the ORF from either the CAP-D3 RE18364 cDNA clone (DGRC) or the RBF1 LD02906 cDNA clone (DGRC) using Pfx polymerase (Invitrogen). The pENTR/D-TOPO Cloning Kit (Invitrogen) was used to clone the ORF into a Gateway entry vector as described in the manufacturer's protocol and at http://www.ciwemb.edu/labs/murphy/Gateway%20vectors.html. The LR Clonase kit (Invitrogen) was then used to recombine the ORF into the pUASt-FHW vector (DGRC) described in detail at the website mentioned above. pUASt-FLAG-HA-RBF1 and pUASt-FLAG-HA-dCAP-D3 vectors were then injected into embryos to create transgenic fly lines expressing the tagged proteins. Mutant flies used as positive controls in infection experiments included the *Imd^1^* strain which was a generous gift from L. Stuart and the *Eater* mutant strain [51]. All flies were maintained at 25°C and placed in vials containing standard dextrose medium.

### Cell culture and RNAi

hTERT-RPE-1 cells were grown in Dulbecco'sModified Essential Medium (DMEM) supplemented with 10% fetal bovine serum (FBS) and 1% penicillin/streptomycin. RNAimax (Invitrogen) was used, according to manufacturer's protocol, to transfect non-targeting, RB, and CAP-D3 specific siRNAs (described in [12]) at final concentrations of 100 nM. Total RNA was harvested 48 hours post transfection and reverse transcribed into cDNA, as described below.

### qRT–PCR

TRIzol (Invitrogen) was used to harvest total RNA from whole flies/specific tissues according to the manufacturer's protocol. After RNA was purified using the Qiagen RNAeasy kit, the Taqman Reverse Transcription kit (Applied Biosystems) was used to reverse transcribe 1.5 µg of RNA into cDNA. qRT-PCR was performed using the Roche Lightcycler 480 to amplify 15 µL reactions containing .5 µL of cDNA, .5 µL of a 10 µM primer mix and 7.5 µL of SYBR Green Master Mix (Roche). All qRT-PCR experiments were performed using three groups of 5 flies per genotype and three independent experiments were performed. Primer sequences are as follows: Rbf1qPCR F1- CTGCAGGGCTACGAGACGTAC, Rbf1qPCR R1 GTGTGCTGGTTCTTCGGCAGG, Rbf2qPCR R1- CTCCCAGTGCTTCTAGCACGC, Rbf2qPCR F1- CGTGAACGCCTTAGAGGTGCC, dCAP-D3 qPCR F3- CGTGCTGTTGCTTTACTTCGGCC, dCAP-D3 qPCR R3- GGCGCATGATGAAGAGCATATCCTG, AttAqPCR F1-GTGGTCCAGTCACAACTGGCG, AttAqPCR R1- CTTGGCATCCAGATTGTGTCTGCC, DroqPCR F1-CACCATCGTTTTCCTGCTGCTTGC, DroqPCR R1-GGTGATCCTCGATGGCCAGTG, AttBqPCR F1- CTCAAAGCGGTCCAGTCACAACTG, AttBqPCR R1- GAATAAATTGGCATGGGCCTCCTGC, Dro4qPCR F1- GTTTGCTCTCCTCGCTGTGGTG, Dro4qPCR R1-GCCCAGCAAGGACCACTGAATC, Dro3qPCR F1- GGCCAACACTGTTTTGGCACGTG, Dro3qPCR R1- GTCCCTCCTCAATGCAGAGACG, Dro2qPCR F1- GTTGTCCTGGCCGCCAATATGG, Dro2qPCR R1- GGACTGCAGTGGCCACTGATATG, DptBqPCR F1- GGACTGGCTTGTGCCTTCTCG, DptBqPCR R1- CAGGGGCACATCAAAATTGGGAGC, DrsqPCR F1-GTACTTGTTCGCCCTCTTCGCTG, DrsqPCR R1- CAGGTCTCGTTGTCCCAGACG, DptqPCR F1- GCTTATCCGATGCCCGACGAC, DptqPCR R1-GTGACCCTGGACTGCAAAGCC, DefqPCR F1- CAAACGCAGACGGCCTTGTCG, DefqPCR R1- AAGCGAGCCACATGCGACCTAC, Dro5qPCR F1- CAAGTTCCTGTACCTCTTCCTGGC, Dro5qPCR R1- CAGGGTCCTCCGTATCTTCCAG, Dro6 qPCR F1-CTTCGCACCAGCATTGCAGCC, Dro6qPCR R1- GAAGGTACAGACCTCCCTGTGC, Dro7qPCR F1- GGCTGCAGTGTCCACTGGTTC, Dro7qPCR R1- CACATGCCGACTGCCTTTCCG, MtkqPCR F1- GATTTTTCTGGCCCTGCTGGGTG, MtkqPCR R1- GGTTGGTTAGGATTGAAGGGCGAC, rp49qPCR F1- TACAGGCCCAAGATCGTGAAG, rp49qPCR R1- GACGCACTCTGTTGTCGATACC, CecCqPCR F1-CAATCGGAAGCCGGTTGGCTG, CecqPCR R1-GCGCAATTCCCAGTCCTTGAATGG, AndqPCR F1- CATTTTGGCCATCAGCGTGGGTC, AndqPCR R1- GGGCTTAGCAAAGCCAATTCCCAC, AttCqPCR F1- GTACTTGGCTCCCTTGCGGTG, AttCqPCR R1- CTTAGGTCCAATCGGGCATCGG, AttDqPCR F1- CCAAGGGAGTTTATGGAGCGGTC, AttDqPCR R1- GCTCTGGAAGAGATTGGCTTGGG, CecA1qPCR F1- CAATCGGAAGCTGGGTGGCTG, CecA1qPCR R1- GGCGGCTTGTTGAGCGATTCC, CecA2qPCR F1- GGACAATCGGAAGCTGGTTGGC, CecA2qPCR R1- GGCCTGTTGAGCGATTCCCAG, CecBqPCR F1- GATTCCGAGGACCTGGATTGAGG, CecBqPCR R1- GGCCATCAGCCTGGGAAACTC, tub84BqPCR F1- GGCAAGGAGATCGTCGATCTGG, tub84BqPCR R1- GACGCTCCATCAGCAGCGAG, hCAP-D3qPCR F1- TCCGGAAGCAGGCCCTCCAG, hCAP-D3qPCR R1- GGACCTGGCTGTCGTCCCCA, hRBqPCR F1- AGCTGTGGGACAGGGTTGTGTC, hRBqPCR R1- CAACCTCAAGAGCGCACGCC, eaterqPCR F1: CTCGTATCGGCTCAGATCTGCAC, eaterqPCR R1: CATCTGAGTGCGGAGCTCCTTAC, IMDqPCR F1- CGAATCCACTGGAGCAACAGCTG, IMDqPCR R1- GTTTCCACGCACTTGGGCGAG, hGAPDHqPCR F1- AGCCTCCCGCTTCGCTCTCT, hGAPDHqPCR R1- CCAGGCGCCCAATACGACCA, orc1qPCR F1- CATCATCCTCAAACACGCGCTGC, orc1qPCR R1- CCCTCGACGAGGCGTAAAAGC, cg5250qPCR F1- GACATTGCCGGAGGTGAAGAGC, cg5250qPCR R1- CTATTCGACTATGTGGTGGGCCTG, dupqPCR F1- GGGTGGCGGTATTTTTGTGGGAG, dupqPCR R1- CAACAGGAAACTCCGCGACGC, mus209qPCR F1- CTTGTCGAAGCCATCGGAACGC, mus209qPCR R1- GGGTCAAGCCACCATCCTGAAG, dnkqPCR F1- CCGCCCCAACCAACAAGAAGC, dnkqPCR R1- CCTCCAGCGTATTGTACATGCCC, RnrSqPCR F1- GAAGAAGGCAAGCACGTGCGAG, RnrSqPCR R1- CCAGTACCACGACATCTGGCAG, dnapoldeltaqPCR F1- CCATCGCCCATTAGCAGAGTCTG, dnapoldeltaqPCR R1- GGAACCTCCAATGGACATGCCAAG, mcm7qPCR F1- CATTGAGCACCGCCTGATGATGG, mcm7qPCR R1- GAGTGCGCCTTCTCTGTGGAC, mcm3qPCR F1- CGAGGTGATGGAACAGGGTCG, mcm3qPCR R1- GAAAGCAGCGAATCCTGCAGTCC, mcm2qPCR F1- GAGATCCCGCAGGACTTGTTGC, mcm2qPCR R1- CAAAAGACTCCTGTCGCAGCTGG, mcm5qPCR F1- CTGGTCTCACGGCTTCGGTTATG, mcm5qPCR R1- GCCACACGATCATCCTCTCGC, dnapolalpha50qPCR F1- CCTTCTACCGTTGGCTATCGTATGG, dnapolalpha50qPCR R1- CAGCTTGGGTATCAAAGCAGAGG, DEFA-1qPCR F1- TGCCCTCTCTGGTCACCCTGC, DEFA-1qPCR R1- GCCTGGAGTGGCTCAGCCTG, DEFB-3qPCR F1- GCGTGGGGTGAAGCCTAGCA, DEFB-3qPCR R1- AGCTGAGCACAGCACACCGG.

### Generation of anti–dCAP-D3 antibody

The rabbit anti–dCAP-D3 YZ834 antibody was generated by Yenzyme Corporation. The antibody was purified using the BIO-RAD Affi-Gel 10 Gel according to manufacturer's protocol.

### Immunofluorescence analysis of cryosections

Adult female flies were cryosectioned (10 µm) and stained as previously described [80]. Primary antibodies included RBF1 (DX2), dCAP-D3 (YZ384), and anti-GFP (Jackson Immunoresearch). Images were obtained using a Zeiss LSM510 Confocal microscope.

### Preprocessing of array data

Nimblegen microarray data were pretreated according to the manufacturer's recommendation and replicate probes were averaged. Affymetrix microarray data was downloaded from array express as raw .CEL files and normalized by robust multi array averaging (RMA)[RMS] before further analysis [81]. The entire set of microarray data can be found in Table S1.

### Hypothesis testing

Differentially expressed genes were identified using a linear model with a moderated T-test [82]. P values were corrected for multiple testing by calculating false discovery rates using the method of Benjamini and Hochberg [83]. Genes with a false discovery rate (FDR)<0.15 and a log2 fold change >0.1 were taken as significant. Gene ontology (GO) annotations were downloaded from FLYBASE [84], and gene ontology terms overrepresented on the lists of differentially expressed genes were identified using a hypergeometric test. P-values from the hypergeometric test were corrected for multiple testing using the same method as for the individual genes and GO-categories with FDR<0.05 were taken as significant.

### Gene clustering analysis

Chromosomal positions of transcription start and stop sites for all genes on the chip were taken from FLYBASE. Genes were counted as clustered if they overlapped, or if the genes lay within 10 000 base pairs of each other. Overall chromosomal clustering for a list of genes was quantified as the number of genes that co-localize according to this criterion. Significance of co-localization was evaluated by comparing to lists of randomly selected genes from the same chip.

### Infection of flies with pathogenic bacteria

*S. aureus* and *P. aeruginosa* bacteria were gifts from L. Stuart. *S. aureus* was grown in a shaking incubator at 37°C, in DIFCO Columbia broth (BD Biosciences) supplemented with 2% NaCl and *P. aeruginosa* was grown in a shaking incubator at 37°C in DIFCO Luria broth (BD Biosciences). Bacteria were inoculated in 10 mL cultures grown overnight. 10^∧4^ bacterial cells were then inoculated into a new 10 mL culture and this was grown to an OD_600 nm_ of 0.5. These cultures were then centrifuged at 3000 rpm in a 1.5 mL eppendorf tube for 5 minutes at 4°C and subsequently washed twice with PBS. After a third centrifugation, PBS wash was removed from the pellet and 25 µL of new PBS was used to resuspend the pellet. Infections were performed as previously described [85]. Specifically, a .25 mm diameter straight stainless steel needle and pin vise (Ted Pella Inc, Redding, CA) were used to infect adult flies. The needle was dipped into the resuspended bacterial pellet and used to prick the thorax of a CO_2_-anesthetized adult fly in a region just underneath where the wing connects to the thorax. Flies were then separated from the needle using a brush and put into fresh vials containing standard dextrose medium with no more than 10 flies per vial.

### Chromatin immunoprecipitation

40 flies per IP were used in all ChIP experiments. Flies were homogenized with a KONTES pellet pestle grinder (Kimble Chase) in 1 mL of buffer A (60 mM KCl, 15 mM NaCl, 4 mM MgCl2, 15 mM HEPES pH 7.6, .5% Triton X-100, .5 mM DTT, EDTA-free protease inhibitors cocktail (Roche)) containing 1.8% formaldehyde. Homogenized flies were incubated at RT for 15 minutes, at which point glycine was added to a concentration of 225 mM. 2–4 mLs of homogenized flies were transferred to 15 mL conical tubes and centrifuged at 4°C for 5 min at 4000 g. Supernatant was discarded and pellets were washed with 3 mL of buffer A. Tubes were centrifuged as described above, supernatant was discarded, and pellets were washed with 3 mL of buffer B (140 mM NaCl, 15 mM HEPES pH 7.6, 1 mM EDTA, .5 mM EGTA, 1% Triton, .5 mM DTT, .1% sodium deoxycholate, EDTA free protease inhibitors cocktail). Tubes were centrifuged as described above, supernatant was discarded, and 500 µL of buffer B+1% SDS per IP was added to each tube. Tubes were rotated at 4°C for 20 min. Samples were then sonicated using the Branson sonifier at a setting of 3, with 8 sonication intervals of 20 seconds interspersed by 10 second breaks. Tubes were centrifuged at 4°C for 5 min at 2000 RPM and 500 µL supernatant was used for each IP. 50 µL of Dynal Protein A beads (Invitrogen) per IP were prepared according to the manufacturer's recommendations. Beads were incubated with anti-FLAG M2 antibody (Sigma) or dCAP-D3 antibody (YZ384) for 2 hours at RT with rotation. Beads were washed according to manufacturer's protocol and added to the diluted chromatin samples which were then incubated at 4°C overnight, with rotation. Samples were centrifuged at 3000 RPM, 4°C for 1 min and washed three times with buffer B+.05% SDS and once with TE. Bound protein was eluted by adding 125 µL of Buffer C (1%SDS, .2% NaCl, TE) to the beads for 30 min at 65°C. Samples were again centrifuged and eluates were harvested and incubated for 4 hours at 65°C to reverse crosslinks. Samples were digested with Proteinase K and RNase A (Sigma), phenol-chloroform extracted, and ethanol precipitated. DNA pellets were dissolved in 105 µL of ddH2O and .5 µL was used per qRT-PCR reaction.

### Survival curves

Flies were collected approximately 5–8 days after eclosure and were infected as described above. Following infection, each group of flies was placed in a new vial of food and monitored for the number of surviving flies at each timepoint. Three experiments were performed, with each experiment including 3 groups of 10 flies per genotype per timepoint. Survival statistics were calculated using a cox proportional hazard model, and hazard ratios with a two sided p-value less than 0.05 were taken as significant.

### Bacterial clearance assays

Flies were anestitized by CO2 inhalation and infected as described above. Following infection, flies were dipped in 95% Ethanol, air dried, and placed into 1.5 mL Eppendorf tubes containing 500 µL of PBS. Flies were homogenized with a Kontes battery powered homogenizer and plastic pestle (USA scientific). The tubes were centrifuged for 2 min at 3000 rpm. Various dilutions were plated onto Columbia CNA with 5% Sheep's Blood Agar (Becton Dickinson and Company). This type of agar contains antibiotics to inhibit growth of organisms other than *Staphylococcus aureus*.

## Supporting Information

Figure S1 dCAP-D3 and RBF1 mutants expressing a transheterozygous combination of alleles retain approximately 15% of wild type protein expression. qRT-PCR (A) and Immunoblots (B) for *rbf1* transcript levels/protein levels and *dCap-D3* transcript levels/protein in wild type (*w^1118^*) and dCAP-D3 transheterozygous mutant (*dCAP-D3^c07081/Δ25^*) or RBF1 transheterozygous mutant (*rbf1^120a/Δ14^*) female flies indicates that mutants retain 10–15% of wild-type protein expression levels. Transcript levels were normalized to *tubulin 84B* mRNA levels and α-tubulin was used as a loading control in B.
(TIF)

Figure S2 RBF1 regulates E2F targets in specific tissues of the adult fly. Ovaries were dissected from female adult flies and cDNA was made from either carcass or ovaries. Top table: qRT-PCR analyses performed on cDNA from ovaries shows that decreased RBF1 expression results in the upregulation of a few E2F targets while decreased dCAP-D3 expression largely has no effect. Bottom table: qRT-PCR analyses performed on cDNA from carcass without ovaries shows that decreased RBF1 expression in the carcass does result in upregulation of many E2F targets, however, dCAP-D3 does not share regulation of these genes with RBF1. Transcript levels were normalized to *tubulin 84B* mRNA levels. All results were significant with p-values≤0.05.
(TIF)

Figure S3 RBF1 and dCAP-D3 antibodies are specific. Immunostaining for dCAP-D3 (A) and RBF1 (B) in cryosections of abdomens of adult female flies expressing dCAP-D3 (A) or RBF1 (B) dsRNA in combination with GFP protein in fat body cells shows the antibodies recognize protein where dsRNAs are not expressed. Flies used in A were of the genotype *yolk-GAL4*, *UAS-GFP/+;+;UAS-dCAP-D3 dsRNA*, and flies used in B were of the genotype *yolk-GAL4*, *UAS-GFP/+;+;UAS-RBF1 dsRNA*.
(TIF)

Figure S4 qRT–PCR analysis of genes adjacent to the *diptericin* locus. qRT–PCR analysis of cDNA from 1) flies expressing driver alone (*yolk-GAL4/+;+;+*), 2) flies expressing *rbf1 dsRNA (yolk-GAL4;+;UAS-rbf1 dsRNA)* in the fat body cells and 3) flies expressing *dCAP-D3 dsRNA (yolk-GAL4;+;UAS-dCAP-D3 dsRNA)* in the fat body cells demonstrates that *CG43070* is also activated by RBF1 and dCAP-D3.
(TIF)

Figure S5 Endogenous dCAP-D3 binds to *CG5250* in a similar pattern as FLAG-HA-dCAP-D3. A) qRT-PCR was performed on cDNA generated from whole female flies (1) expressing yolk-GAL4 driver alone (*yolk-GAL4/+; +;+*) or (2) exhibiting acute, fat body specific knockdown of dCAP-D3 (*yolk-GAL4/+; +/UAS-dCAP-D3 dsRNA/+*). Transcript levels for genes surrounding *CG5250* indicate that *CG5250* is the only gene in the locus that is significantly regulated by dCAP-D3. B) Graphic representation of the *CG5250* locus on which ChIP for dCAP-D3 in both the whole adult and the adult fat body was performed. Relative positions of primer sets used are listed under the diagram of the locus. *CG5250* is highlighted in red since it is repressed by dCAP-D3 in the whole adult fly. C) Chromatin immunoprecipitation for FLAG protein in female adult flies expressing FLAG-HA-dCAP-D3 in the fat body (*yolk-GAL4/+; +; UAS-FLAG-HA-dCap-D3/+*) shows that the *CG5250* locus is a direct target of dCAP-D3. ChIP signal corresponding to FLAG-HA-dCAP-D3 binding in the absence of *Staphylococcus aureus* infection is colored in burgundy. ChIP signal corresponding to FLAG-HA-dCAP-D3 binding two and four hours after *S. aureus* infection is colored in blue and yellow, respectively. D) ChIP for endogenous dCAP-D3 in whole adult flies at the *CG5250* locus demonstrates the dCAP-D3 binding pattern is identical to the pattern exhibited specifically in the fat body.
(TIF)

Figure S6 Confirmation of rbf1 and rbf2 transcript knockdown in flies expressing RBF2 or RBF1 and RBF2 dsRNAs. qRT-PCR was performed on cDNAs from flies deficient for RBF2 alone or deficient for a combination of both RBF1 and RBF2.
(TIF)

Figure S7 RBF2 does not regulate AMP induction following bacterial infection. Adult female flies expressing RBF2 (turquoise) or a combination of RBF1 and RBF2 (black) dsRNAs under the control of *yolk-GAL4* were infected with the Gram positive bacteria, *Staphylococcus aureus*. A) qRT-PCR analyses for transcript levels of the *Drosomycin* AMP gene in these flies show that control flies expressing GFP dsRNAs under the control of *yolk-GAL4* (green) undergo a large induction of AMPs at 8–24 hours post-infection. Flies expressing RBF2 dsRNA or a combination of RBF1 and RBF2 dsRNAs show no significant, repeated changes in transcript levels upon comparison to control flies. B) qRT-PCR analyses for transcript levels of the *Diptericin* AMP gene in these flies show that control flies expressing GFP dsRNAs under the control of *yolk-GAL4* (green) undergo a large induction of AMPs at 8–24 hours post-infection. Flies expressing RBF2 dsRNA or a combination of RBF1 and RBF2 dsRNAs exhibit a significant decrease in basal transcript levels in the majority of experiments, but do not exhibit significant changes in transcript levels following infection. Three independent experiments are shown and results for each experiment are the average of three sets of five infected adults. The inset boxes in the upper right corner of each graph are a larger representation of the 0 hour timepoint and therefore depict basal transcription levels. Asterisks emphasize statistical significance (p≤0.05) as determined by a students paired t-test.
(TIF)

Figure S8 RBF2 deficiency in the fat body does not significantly affect survival following bacterial infection. Adult female flies expressing RBF2 (turquoise) dsRNAs or a combination of RBF1 and RBF2 dsRNAs (black) under the control of *yolk-GAL4* were infected with the Gram positive bacterium, *Staphylococcus aureus* (A) or the Gram negative bacterium, *Pseudomonas aeruginosa* (B). Flies expressing GFP dsRNAs under the control of *yolk-GAL4* (green) were used as “wild-type” controls. Eater mutants which are defective in phagocytosis (blue) or flies expressing IMD dsRNAs which are compromised in the Gram negative arm of the innate immune signaling pathway (yellow) were used as positive controls. Results demonstrate that flies expressing reduced levels of RBF2 or reduced levels of both RBF1 and RBF2 in the fat body cells do not significantly and repeatedly affect survival times in response to either type of infection upon comparison to wild type controls. Three independent experiments are depicted with results of each experiment shown as the average of three sets of 10 infected adults per genotype. Results are presented as cox regression models with statistical significance (p≤0.05) represented as shaded areas above and below the curves. These experiments were also performed using a sterile needle dipped in PBS to rule out death as a result of wounding and survival curves matched those of yolk-GAL4 expressing flies (data not shown).
(TIF)

Figure S9 Regulation of AMP genes by RB and CAP-D3 is conserved in human cells. A) RPE-1 cells were transfected with (1) non-targeting Control siRNA, (2) pRB siRNA or (3)CAP-D3 siRNAs. qRT-PCR analyses were performed on cDNAs generated from cellular RNA collected 48 hours post transfection and results show that RB and CAP-D3 are significantly decreased. B) qRT-PCR for AMPs in cells described in A shows that pRB or dCAP-D3 deficiency results in significant decreases in basal levels of two human AMP genes.
(TIF)

Table S1 Microarray data from multiple samples of wild type cDNA, dCAP-D3 mutant cDNA, and RBF1 mutant cDNA. Experiments were performed using Nimblegen 385 k whole genome arrays. A detailed description of the information found in each column is provided to the right of the table.
(XLS)

Table S2 Analysis of clustering frequencies among dCAP-D3 and/or RBF1 regulated target genes. A detailed description of the information found in each column is provided to the right of the table. Numbers within individual columns are arbitrary and designate genes present within the same cluster; they do not indicate any information about the strength of their misregulation in mutant flies.
(XLS)

Table S3 List of significant Gene Ontology categories represented by the total number of shared RBF1/dCAP-D3 target genes in the adult fly. Column C lists the Gene Ontology (GO) term. Column D lists the term name associated with the GO category. Column E lists the total number of significant genes found in this GO category. Column F lists the total number of genes found in this GO category. Column G lists the P values associated with the GO category. Column H lists the actual Flybase Gene Numbers associated with the microarray genes found in the GO category.
(XLS)

Table S4 dCAP-D3/RBF1 do not regulate the majority of genes previously reported to be involved in phagocytosis. Microarray data from dCAP-D3 mutant adult flies vs. wild type flies (Table S1) was analyzed for changes to transcript levels of 19 genes previously reported to be involved in phagocytosis (Column A). Only two genes were shown to be misregulated in the mutant flies (Columns B and C).
(XLS)

Table S5 dCAP-D3/RBF1 do not regulate the majority of genes previously reported to be involved in coagulation and melanization. Microarray data from dCAP-D3 mutant adult flies vs. wild type flies (Table S1) was analyzed for changes to transcript levels of 18 genes previously reported to be involved in coagulation and melanization (Column A). Only one gene was shown to be misregulated in the mutant flies (Columns B and C).
(XLS)
