# Supplementary figures and images for: A Shared Role for RBF1 and dCAP-D3 in the Regulation of Transcription with Consequences for Innate Immunity
Source: PLoS Genet. 2012 Apr 5;8(4):e1002618. doi: 10.1371/journal.pgen.1002618 (PMC3320600; doi:10.1371/journal.pgen.1002618)

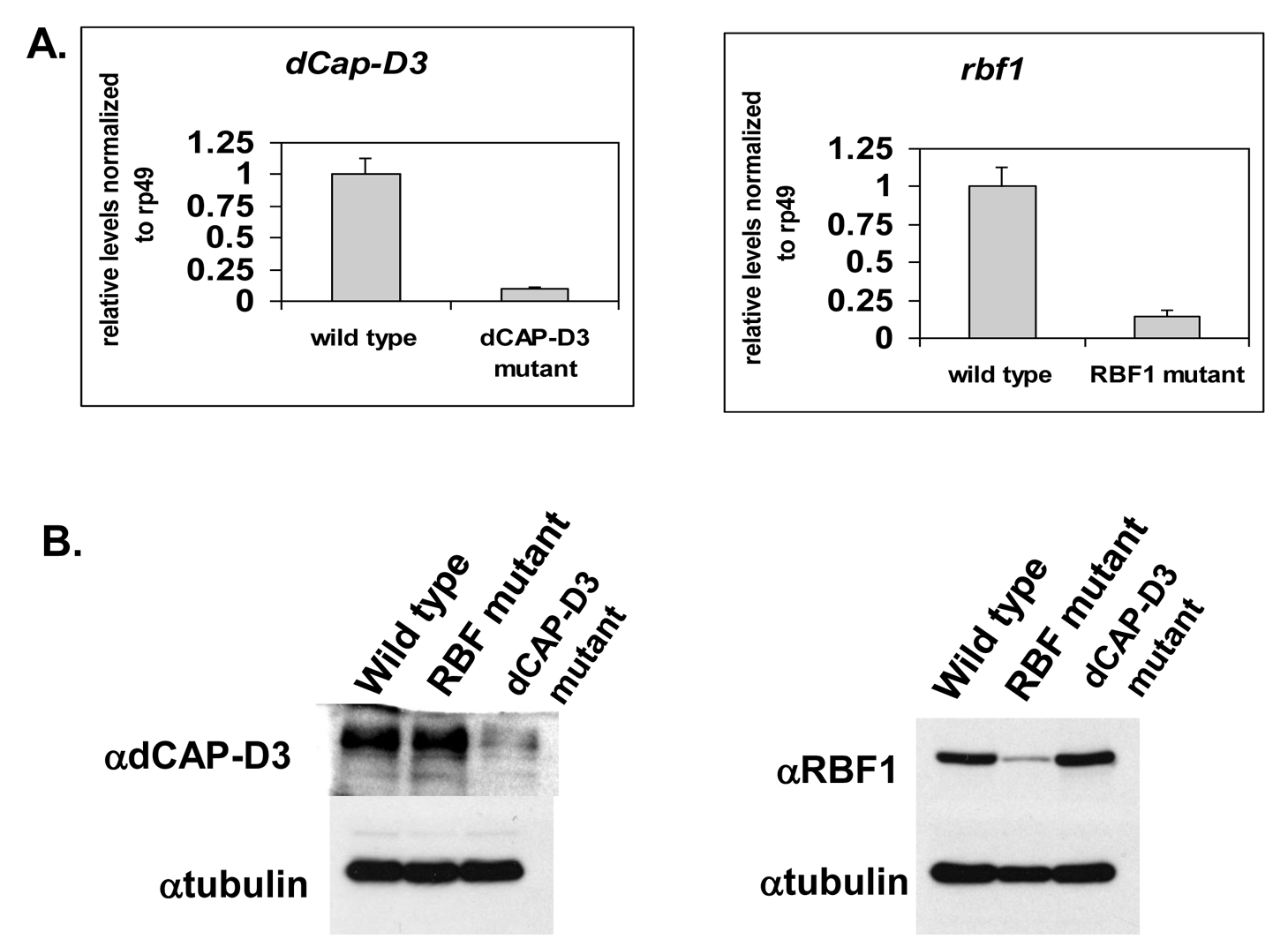

Supplement: Figure S1 — dCAP-D3 and RBF1 mutants expressing a transheterozygous combination of alleles retain approximately 15% of wild type protein expression. qRT-PCR (A) and Immunoblots (B) for rbf1 transcript levels/protein levels and dCap-D3 transcript levels/protein in wild type (w1118) and dCAP-D3 transheterozygous mutant (dCAP-D3c07081/Δ25) or RBF1 transheterozygous mutant (rbf1120a/Δ14) female flies indicates that mutants retain 10–15% of wild-type protein expression levels. Transcript levels were normalized to tubulin 84B mRNA levels and α-tubulin was used as a loading control in B. (TIF) [file pgen.1002618.s001.tif]

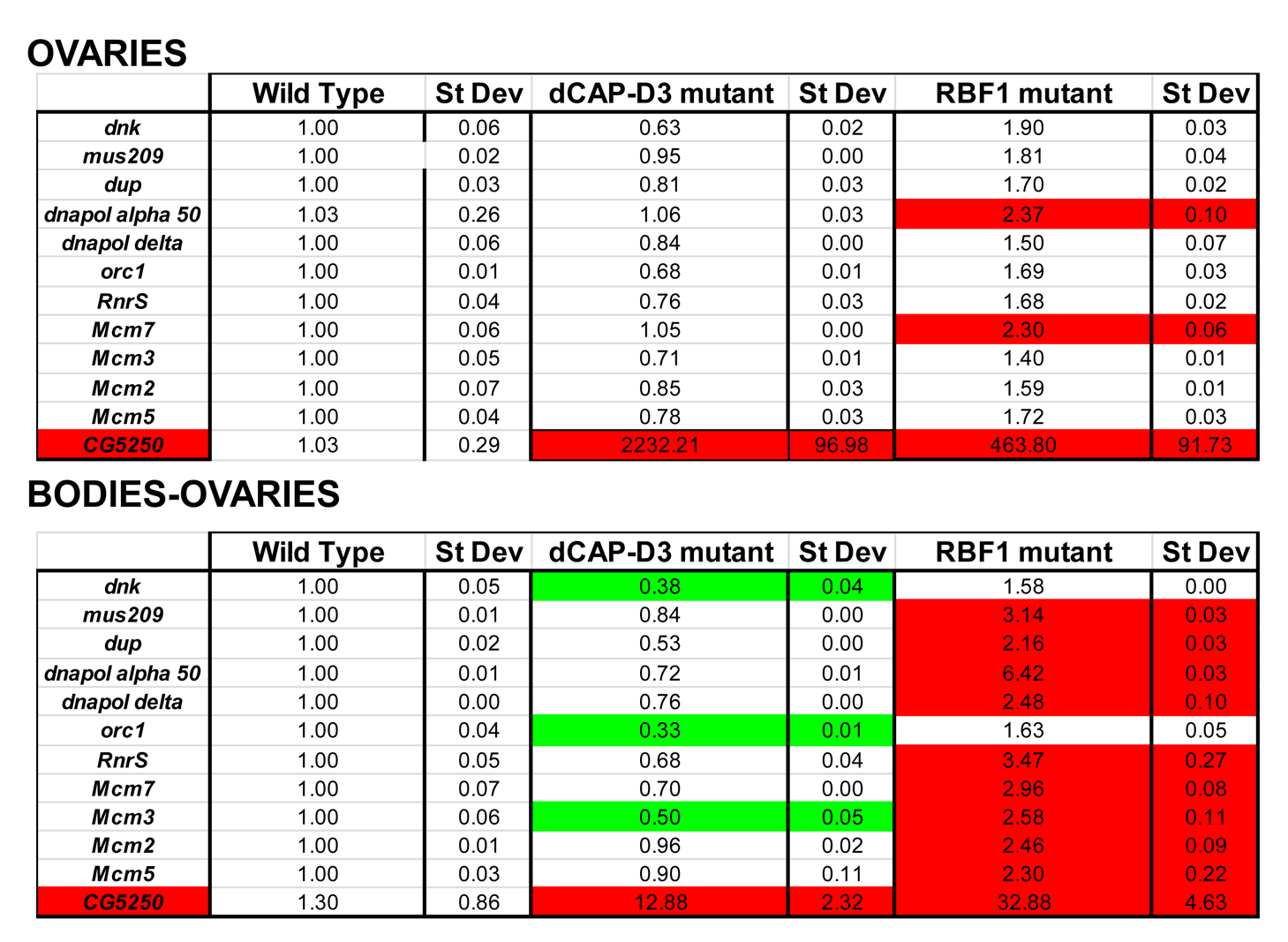

Supplement: Figure S2 — RBF1 regulates E2F targets in specific tissues of the adult fly. Ovaries were dissected from female adult flies and cDNA was made from either carcass or ovaries. Top table: qRT-PCR analyses performed on cDNA from ovaries shows that decreased RBF1 expression results in the upregulation of a few E2F targets while decreased dCAP-D3 expression largely has no effect. Bottom table: qRT-PCR analyses performed on cDNA from carcass without ovaries shows that decreased RBF1 expression in the carcass does result in upregulation of many E2F targets, however, dCAP-D3 does not share regulation of these genes with RBF1. Transcript levels were normalized to tubulin 84B mRNA levels. All results were significant with p-values≤0.05. (TIF) [file pgen.1002618.s002.tif]

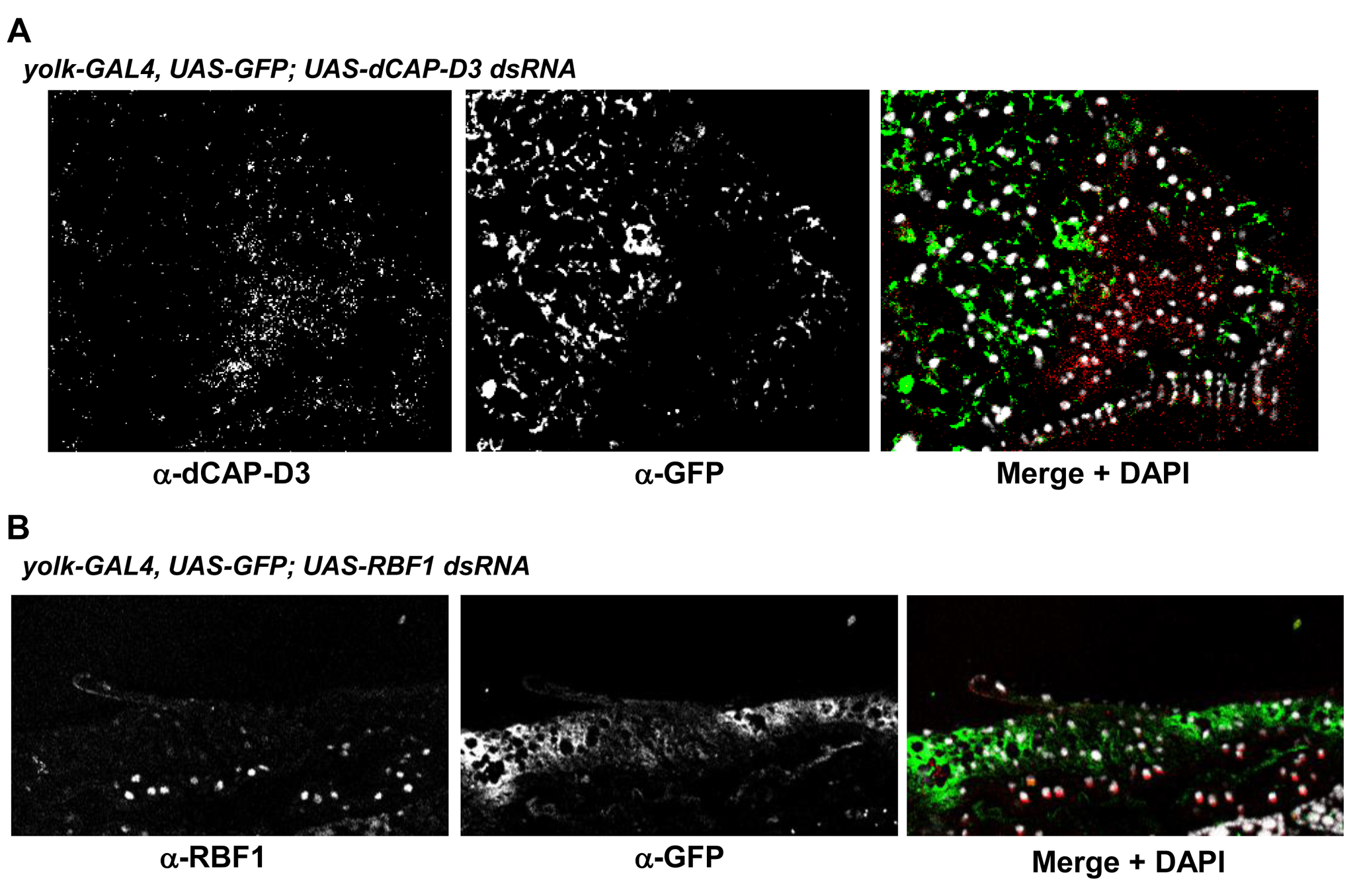

Supplement: Figure S3 — RBF1 and dCAP-D3 antibodies are specific. Immunostaining for dCAP-D3 (A) and RBF1 (B) in cryosections of abdomens of adult female flies expressing dCAP-D3 (A) or RBF1 (B) dsRNA in combination with GFP protein in fat body cells shows the antibodies recognize protein where dsRNAs are not expressed. Flies used in A were of the genotype yolk-GAL4, UAS-GFP/+;+;UAS-dCAP-D3 dsRNA, and flies used in B were of the genotype yolk-GAL4, UAS-GFP/+;+;UAS-RBF1 dsRNA. (TIF) [file pgen.1002618.s003.tif]

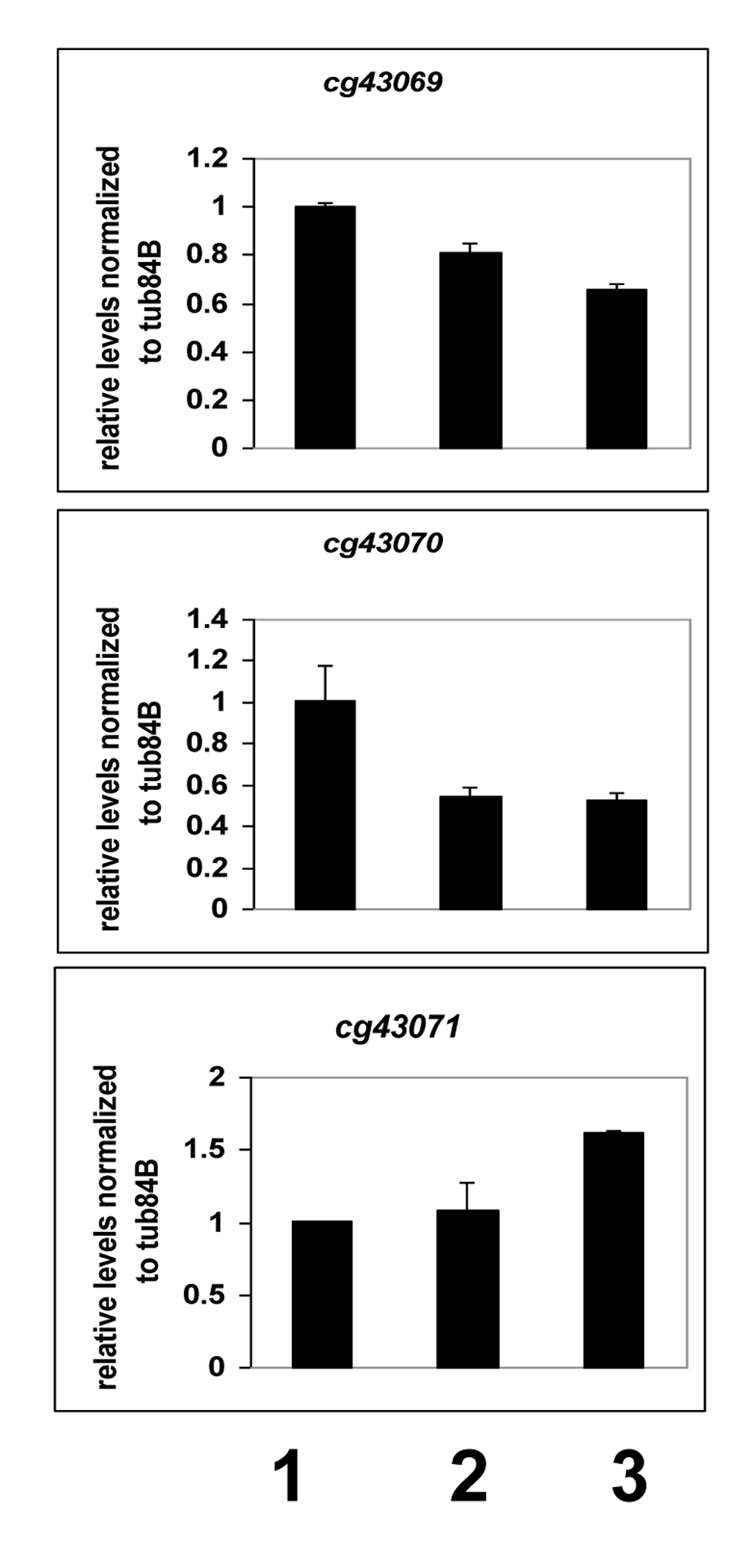

Supplement: Figure S4 — qRT–PCR analysis of genes adjacent to the diptericin locus. qRT–PCR analysis of cDNA from 1) flies expressing driver alone (yolk-GAL4/+;+;+), 2) flies expressing rbf1 dsRNA (yolk-GAL4;+;UAS-rbf1 dsRNA) in the fat body cells and 3) flies expressing dCAP-D3 dsRNA (yolk-GAL4;+;UAS-dCAP-D3 dsRNA) in the fat body cells demonstrates that CG43070 is also activated by RBF1 and dCAP-D3. (TIF) [file pgen.1002618.s004.tif]

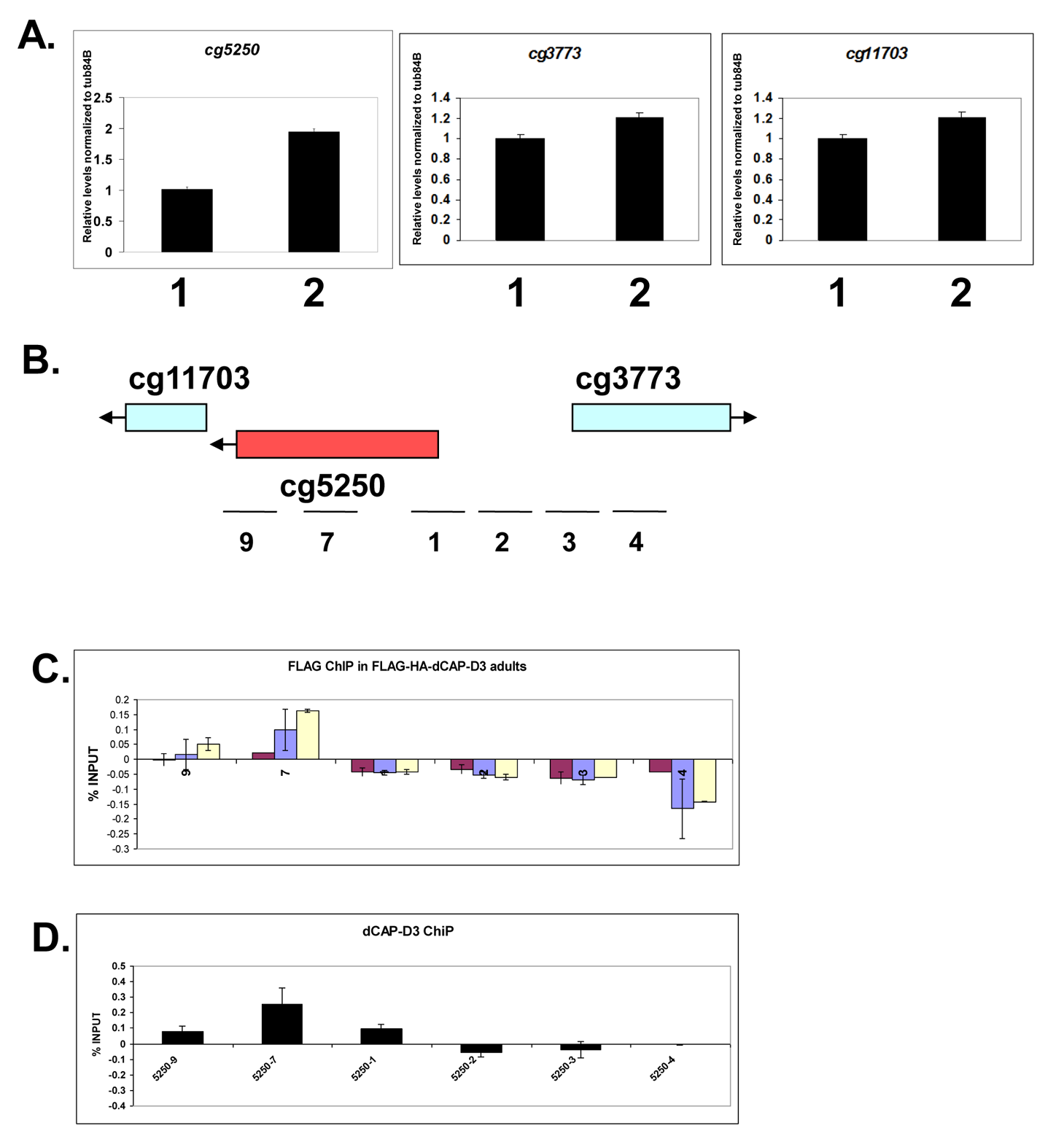

Supplement: Figure S5 — Endogenous dCAP-D3 binds to CG5250 in a similar pattern as FLAG-HA-dCAP-D3. A) qRT-PCR was performed on cDNA generated from whole female flies (1) expressing yolk-GAL4 driver alone (yolk-GAL4/+; +;+) or (2) exhibiting acute, fat body specific knockdown of dCAP-D3 (yolk-GAL4/+; +/UAS-dCAP-D3 dsRNA/+). Transcript levels for genes surrounding CG5250 indicate that CG5250 is the only gene in the locus that is significantly regulated by dCAP-D3. B) Graphic representation of the CG5250 locus on which ChIP for dCAP-D3 in both the whole adult and the adult fat body was performed. Relative positions of primer sets used are listed under the diagram of the locus. CG5250 is highlighted in red since it is repressed by dCAP-D3 in the whole adult fly. C) Chromatin immunoprecipitation for FLAG protein in female adult flies expressing FLAG-HA-dCAP-D3 in the fat body (yolk-GAL4/+; +; UAS-FLAG-HA-dCap-D3/+) shows that the CG5250 locus is a direct target of dCAP-D3. ChIP signal corresponding to FLAG-HA-dCAP-D3 binding in the absence of Staphylococcus aureus infection is colored in burgundy. ChIP signal corresponding to FLAG-HA-dCAP-D3 binding two and four hours after S. aureus infection is colored in blue and yellow, respectively. D) ChIP for endogenous dCAP-D3 in whole adult flies at the CG5250 locus demonstrates the dCAP-D3 binding pattern is identical to the pattern exhibited specifically in the fat body. (TIF) [file pgen.1002618.s005.tif]

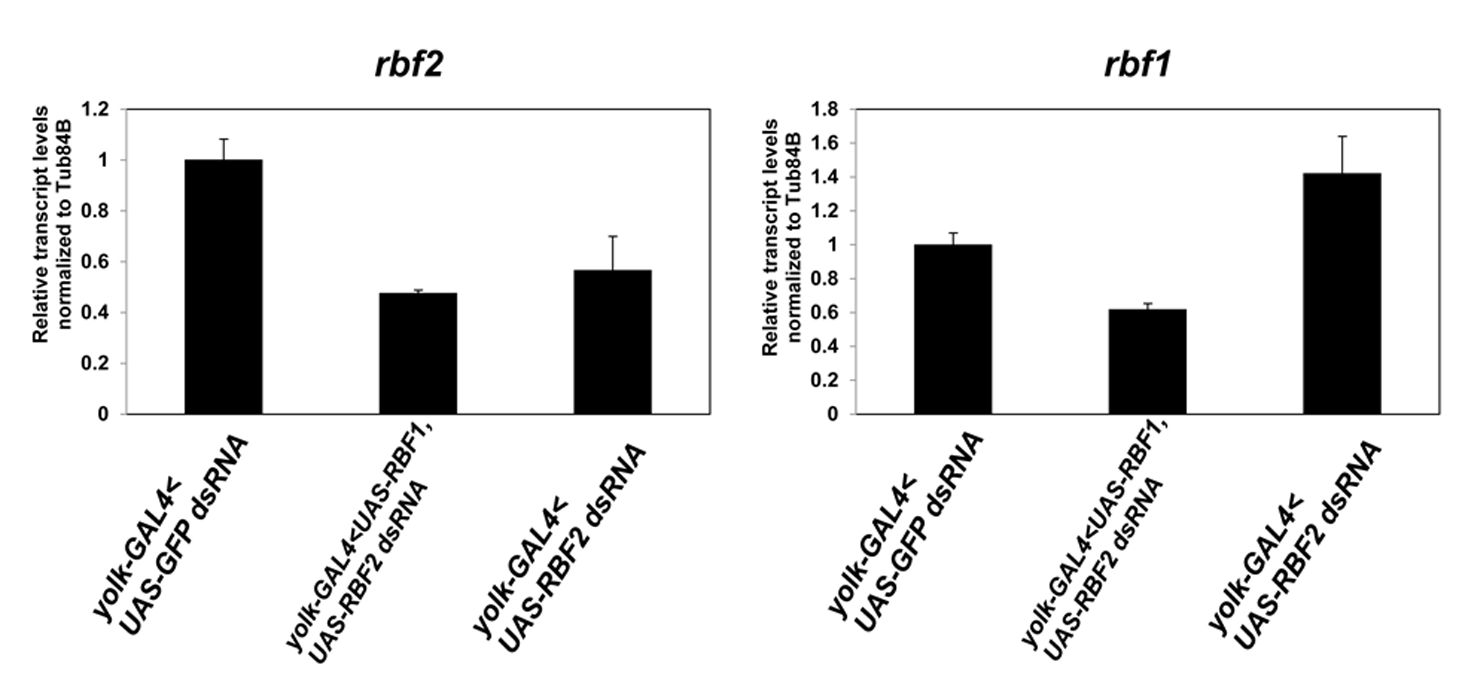

Supplement: Figure S6 — Confirmation of rbf1 and rbf2 transcript knockdown in flies expressing RBF2 or RBF1 and RBF2 dsRNAs. qRT-PCR was performed on cDNAs from flies deficient for RBF2 alone or deficient for a combination of both RBF1 and RBF2. (TIF) [file pgen.1002618.s006.tif]

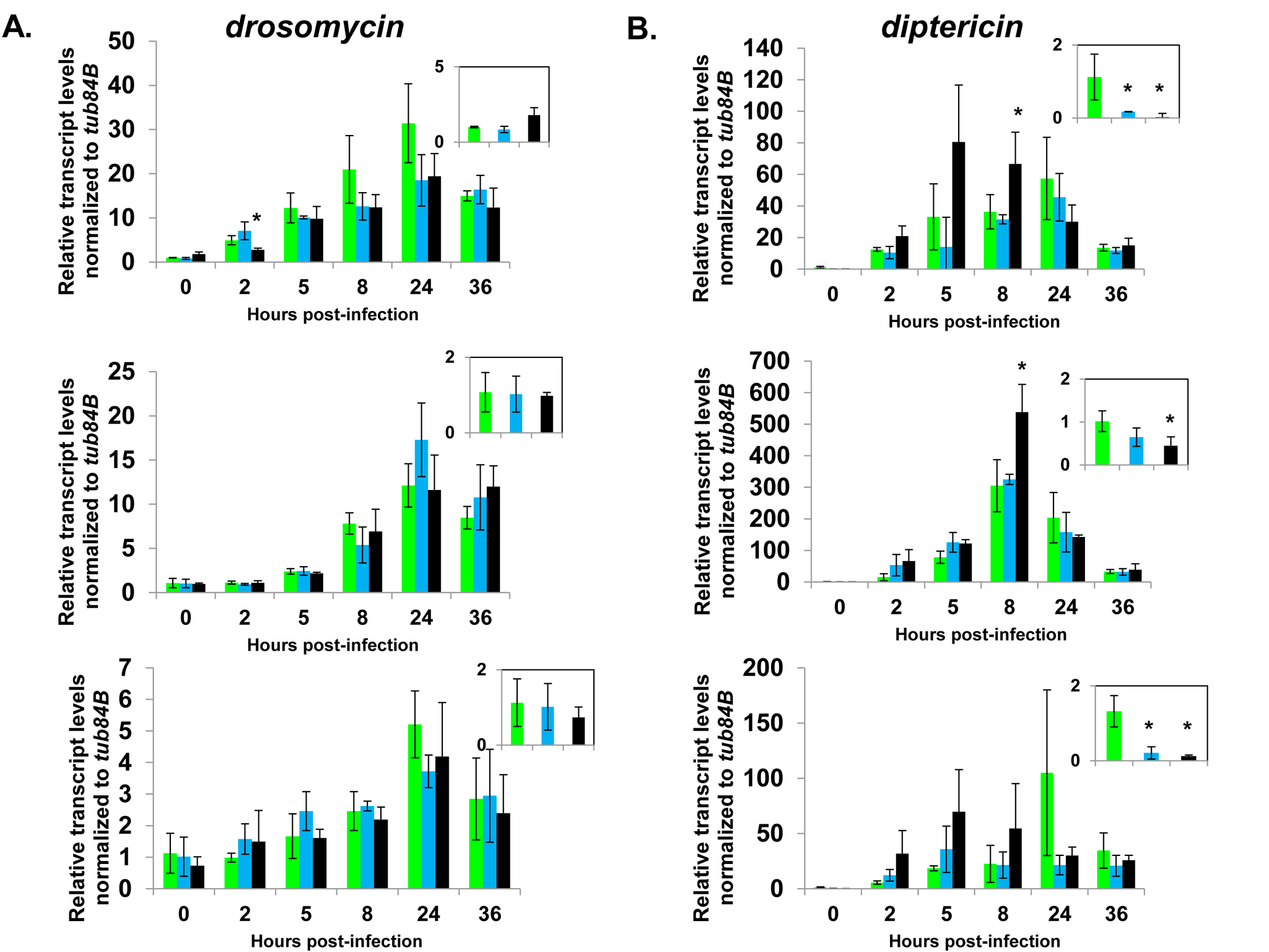

Supplement: Figure S7 — RBF2 does not regulate AMP induction following bacterial infection. Adult female flies expressing RBF2 (turquoise) or a combination of RBF1 and RBF2 (black) dsRNAs under the control of yolk-GAL4 were infected with the Gram positive bacteria, Staphylococcus aureus. A) qRT-PCR analyses for transcript levels of the Drosomycin AMP gene in these flies show that control flies expressing GFP dsRNAs under the control of yolk-GAL4 (green) undergo a large induction of AMPs at 8–24 hours post-infection. Flies expressing RBF2 dsRNA or a combination of RBF1 and RBF2 dsRNAs show no significant, repeated changes in transcript levels upon comparison to control flies. B) qRT-PCR analyses for transcript levels of the Diptericin AMP gene in these flies show that control flies expressing GFP dsRNAs under the control of yolk-GAL4 (green) undergo a large induction of AMPs at 8–24 hours post-infection. Flies expressing RBF2 dsRNA or a combination of RBF1 and RBF2 dsRNAs exhibit a significant decrease in basal transcript levels in the majority of experiments, but do not exhibit significant changes in transcript levels following infection. Three independent experiments are shown and results for each experiment are the average of three sets of five infected adults. The inset boxes in the upper right corner of each graph are a larger representation of the 0 hour timepoint and therefore depict basal transcription levels. Asterisks emphasize statistical significance (p≤0.05) as determined by a students paired t-test. (TIF) [file pgen.1002618.s007.tif]

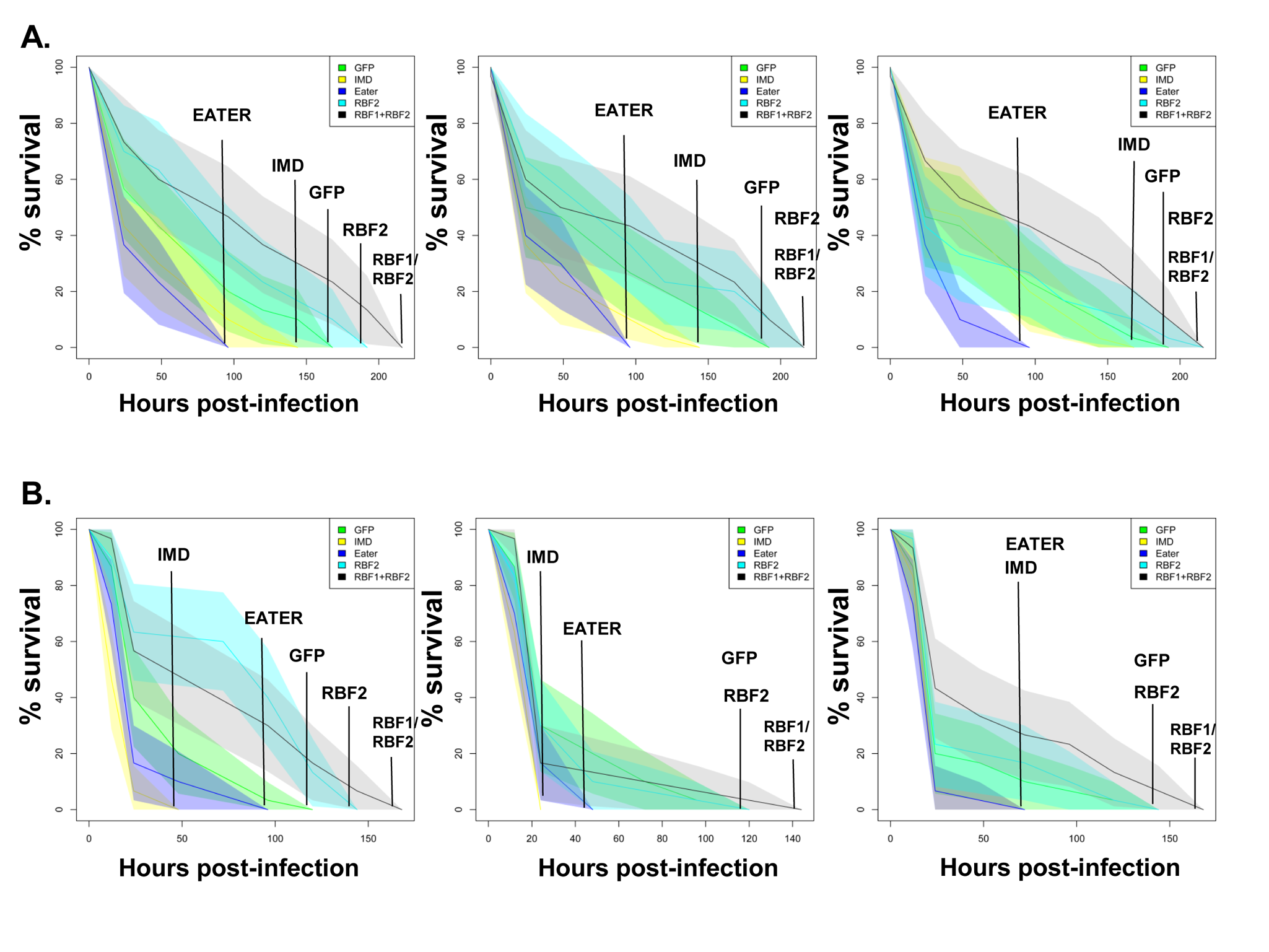

Supplement: Figure S8 — RBF2 deficiency in the fat body does not significantly affect survival following bacterial infection. Adult female flies expressing RBF2 (turquoise) dsRNAs or a combination of RBF1 and RBF2 dsRNAs (black) under the control of yolk-GAL4 were infected with the Gram positive bacterium, Staphylococcus aureus (A) or the Gram negative bacterium, Pseudomonas aeruginosa (B). Flies expressing GFP dsRNAs under the control of yolk-GAL4 (green) were used as “wild-type” controls. Eater mutants which are defective in phagocytosis (blue) or flies expressing IMD dsRNAs which are compromised in the Gram negative arm of the innate immune signaling pathway (yellow) were used as positive controls. Results demonstrate that flies expressing reduced levels of RBF2 or reduced levels of both RBF1 and RBF2 in the fat body cells do not significantly and repeatedly affect survival times in response to either type of infection upon comparison to wild type controls. Three independent experiments are depicted with results of each experiment shown as the average of three sets of 10 infected adults per genotype. Results are presented as cox regression models with statistical significance (p≤0.05) represented as shaded areas above and below the curves. These experiments were also performed using a sterile needle dipped in PBS to rule out death as a result of wounding and survival curves matched those of yolk-GAL4 expressing flies (data not shown). (TIF) [file pgen.1002618.s008.tif]

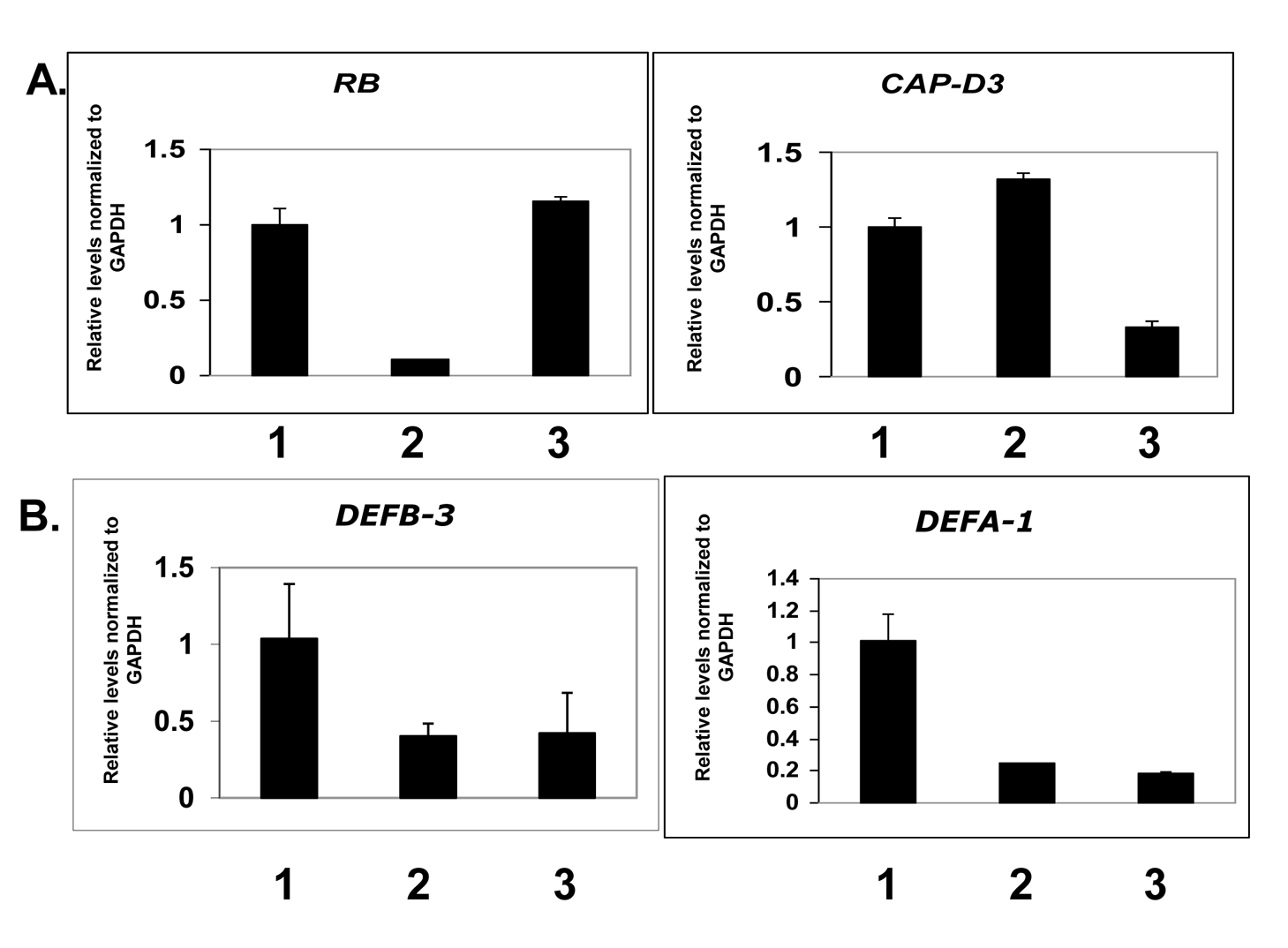

Supplement: Figure S9 — Regulation of AMP genes by RB and CAP-D3 is conserved in human cells. A) RPE-1 cells were transfected with (1) non-targeting Control siRNA, (2) pRB siRNA or (3)CAP-D3 siRNAs. qRT-PCR analyses were performed on cDNAs generated from cellular RNA collected 48 hours post transfection and results show that RB and CAP-D3 are significantly decreased. B) qRT-PCR for AMPs in cells described in A shows that pRB or dCAP-D3 deficiency results in significant decreases in basal levels of two human AMP genes. (TIF) [file pgen.1002618.s009.tif]
